# Biochemical and genetic functional dissection of the P38 viral suppressor of RNA silencing

**DOI:** 10.1261/rna.060434.116

**Published:** 2017-05

**Authors:** Taichiro Iki, Marie-Aude Tschopp, Olivier Voinnet

**Affiliations:** Department of Biology, Swiss Federal Institute of Technology (ETH), 8092 Zürich, Switzerland

**Keywords:** ARGONAUTE (AGO), Dicer-like (DCL), viral suppressor of RNA silencing (VSR), P38, *Turnip crinkle virus* (TCV), *Nicotiana tabacum* BY-2 cell lysate (BYL)

## Abstract

Phytoviruses encode viral suppressors of RNA silencing (VSRs) to counteract the plant antiviral silencing response, which relies on virus-derived small interfering (si)RNAs processed by Dicer RNaseIII enzymes and subsequently loaded into ARGONAUTE (AGO) effector proteins. Here, a tobacco cell-free system was engineered to recapitulate the key steps of antiviral RNA silencing and, in particular, the most upstream double-stranded (ds)RNA processing reaction, not kinetically investigated thus far in the context of plant VSR studies. Comparative biochemical analyses of distinct VSRs in the reconstituted assay showed that in all cases tested, VSR interactions with siRNA duplexes inhibited the loading, but not the activity, of antiviral AGO1 and AGO2. Turnip crinkle virus P38 displayed the additional and unique property to bind both synthetic and RNA-dependent-RNA-polymerase-generated long dsRNAs, and inhibited the processing into siRNAs. Single amino acid substitutions in P38 could dissociate dsRNA-processing from AGO-loading inhibition in vitro and in vivo, illustrating dual-inhibitory strategies discriminatively deployed within a single viral protein, which, we further show, are bona fide suppressor functions that evolved independently of the conserved coat protein function of P38.

## INTRODUCTION

Eukaryotic organisms use RNA silencing to regulate development, stress responses, defense against pathogens, and protection of genomic integrity (Bologna and Voinnet 2014). In plants and invertebrates, antiviral defense relies upon RNA silencing pathway components (Pumplin and Voinnet 2013) and, as a counter-defensive strategy, viral suppressors of RNA silencing (VSRs) have been evolved independently by diverse virus genera (Csorba et al. 2015).

In RNA silencing, RNaseIII family enzymes, including Dicer-like proteins (DCLs) in plants, mediate the processing from longer double-stranded RNA (dsRNA) precursors of small RNA (sRNA) duplexes bearing diagnostic 2-nt 3′ overhangs and 5′-monophosphates. In *Arabidopsis thaliana*, four DCLs display specialized functions by producing specific sRNA products (Bologna and Voinnet 2014). The precise processing of microRNAs (miRNAs) from imperfect, fold-back endogenous transcripts is mostly dependent on DCL1, the activity of which is aided by the RNA-binding partner Hyponastic Leaves 1 (HYL1) and Serrate (Kurihara et al. 2006; Dong et al. 2008; Zhu et al. 2013). The other three DCLs participate in the production of small interfering RNAs (siRNAs). During post-transcriptional gene silencing (PTGS), DCL4 cooperates with dsRNA binding protein 4 (DRB4) to produce 21-nt siRNA duplexes (Nakazawa et al. 2007; Fukudome et al. 2011), while DCL2 produces 22-nt siRNA species acting mostly redundantly with the products of DCL4 (Parent et al. 2015). DCL3 mediates processing of 24-nt siRNAs that promote RNA-directed DNA methylation (RdDM), potentially resulting in transcriptional gene silencing (TGS) (Blevins et al. 2015). sRNA duplexes processed by DCLs are then loaded onto ARGONAUTE proteins (AGOs), in which the selective retention of sRNA guide-strands and elimination of passenger-strands lead to mature RNA-induced silencing complexes (RISCs) that recognize target RNAs containing sequences complementary to the guide strand small RNAs, and mediate their endonucleolytic cleavage and/or translational repression (Baumberger and Baulcombe 2005; Brodersen et al. 2008; Iki et al. 2010; Iwakawa and Tomari 2013). RNA-induced transcriptional silencing complexes formed with DCL3-dependent 24-nt siRNAs mediate RdDM (Ye et al. 2012).

In *Arabidopsis*, resistance to RNA viruses—the largest class of plant viruses—is associated with PTGS pathways in which virus-derived siRNAs (vsiRNAs) are generated through the hierarchical action of primarily DCL4 and DCL2 (Deleris et al. 2006). Potent substrates for vsiRNA production include viral fold-back RNA structures and long dsRNAs synthesized by viral RNA-dependent RNA polymerases (RDRs) as part of the replication process, and by additional activities of host-encoded RDRs including, chiefly, *Arabidopsis* RDR6, RDR1 and, to some extent, RDR2 (Garcia-Ruiz et al. 2010; Wang et al. 2010; Csorba et al. 2015). Antiviral PTGS also requires the cooperative and distinctive functions of AGO1 and AGO2 in *Arabidopsis*, while AGO5, AGO7, and AGO10 have also been shown to play roles under some circumstances (Qu et al. 2008; Wang et al. 2011; Zhang et al. 2012; Garcia-Ruiz et al. 2015).

Our current understanding of viral suppression of RNA silencing shows that binding of virus-derived siRNA is a conserved property displayed by VSRs from diverse viral genera (Csorba et al. 2015). As perhaps the best-characterized VSR, the tombusviral P19 protein acts as dimeric molecular caliper that measures the length of dsRNAs and sequesters siRNA duplexes in a size-selective and sequence-independent manner (Vargason et al. 2003; Ye et al. 2003). The Helper component-protease (HC-Pro) encoded by potyviruses such as *Turnip mosaic virus* (TuMV) and *Potato virus* Y (PVY), also sequesters siRNA duplexes by sensing the sizes, like P19, and also recognizes the 2-nt 3′ overhang diagnostic of DCL-dependent products (Lakatos et al. 2006). Consistent with an siRNA-sequestering mode of action, both P19 and HC-Pro were shown to prevent *Drosophila* AGO2 RISC loading and/or activity in a heterologous fly embryo extract (Lakatos et al. 2006).

Besides these two examples, size-independent interaction with dsRNA has been described for many VSRs including *Turnip crinkle virus* (TCV) P38 (Mérai et al. 2006), *Tomato aspermy virus* 2b (Chen et al. 2008), and *Pothos latent virus* (PoLV) P14 (Mérai et al. 2005), but whether dsRNA binding is indeed genetically required for VSR function has remained unaddressed in most cases, especially since proteins from RNA viruses may display natural affinity to dsRNA as part of replication or structural functions independent of RNA silencing suppression (for review, see Pumplin and Voinnet 2013). For instance, earlier studies have shown the reduced accumulation of 21-nt siRNA species upon expression of TCV P38, consistent with a model in which P38 might antagonize dsRNA processing by binding long dsRNA (Qu et al. 2003; Deleris et al. 2006). However, TCV P38 functions not only as a VSR but also as a coat protein (CP) encapsidating virion RNA (Hogle et al. 1986; Bakker et al. 2012), which may underpin its affinity to dsRNA in a manner unrelated to silencing suppression. Mérai et al. (2006) have shown that transient ectopic expression of P38 strongly reduces hairpin-derived siRNA accumulation in vivo and also stabilizes hairpin dsRNA transcripts. Although this result could be interpreted as evidence that P38 directly inhibits dicing of long dsRNA, it could equally be that P38 binding to long dsRNA is in fact unrelated to its VSR activity and that, rather, siRNA loading into RISC—a step not investigated in the study—is antagonized by P38, leading to siRNA destabilization in vivo. Further consistent with the idea that long dsRNA binding by some VSRs might not be relevant to their function, PoLV P14, despite its strong affinity for dsRNA in vivo and in vitro, prevents accumulation of hairpin-derived siRNAs without stabilizing hairpin dsRNA transcripts when expressed ectopically (Mérai et al. 2005).

Thus, it remains generally unknown if, and how, phytovirus-encoded VSRs exhibit direct inhibitory activities against the Dicer-mediated dsRNA processing step of antiviral RNAi, and, if so, whether the affinity of VSRs for long dsRNA is indeed relevant to this function. Addressing these and other questions has been hampered by the lack of a suitable and universal plant biochemical platform in which the effects of VSRs and mutant derivatives can be assessed in parallel and systematically against each major reconstructed step of the PTGS antiviral pathway, including the most upstream and least characterized dsRNA-processing phase. Recently, a lysate of vacuole-free protoplast from *Nicotiana tabacum* BY-2 cells (BYL), which displays efficient in vitro translation activity, was successfully applied to recapitulate RNA virus replication (Komoda et al. 2004), RISC loading with exogenous sRNAs (Iki et al. 2010, 2012; Ye et al. 2012; Endo et al. 2013), RISC-mediated translational repression by miRNAs (Iwakawa and Tomari 2013) and to show that the stabilization of RISC-cleaved fragments is a critical step for secondary siRNA production by RDRs (Yoshikawa et al. 2013). A recent study has advanced the BYL-based system by demonstrating the antiviral activity of RISC and the suppressor function of *Tomato bushy stunt virus* (TBSV) P19 in the recapitulated TBSV replication (Schuck et al. 2013).

Here, we have reconstructed the key steps of antiviral PTGS in the BYL to systematically investigate the effects of five VSRs from unrelated RNA viruses on each of these steps, focusing in particular on the previously uncharacterized Dicer-mediated dsRNA processing reaction. While all VSRs tested were found to inhibit the loading, but not the activity, of AGO1/2-RISC, P38 could additionally and uniquely inhibit dsRNA processing recapitulated in BYL using exogenous dsRNA as well as dsRNA synthesized by host RDR activities. We thus conducted further experiments on P38, using previously characterized loss-of-function mutant alleles of P38, novel alleles obtained by directed mutagenesis, as well as natural variants of the protein. Our investigations in the BYL-based system allowed us to conclude that (i) dsRNA binding by P38 is required for the processing inhibition, (ii) siRNA binding and RISC-loading inhibition are essential and tightly coupled steps of TCV P38 VSR function, and (iii) inhibition of RISC loading and dsRNA processing are recently acquired properties of TCV P38 and can be uncoupled genetically, leading us to propose a dual-activity model for the silencing suppression mediated by this protein. Further analysis in vivo confirmed that binding to long dsRNA is effectively required for dsRNA-processing inhibition and is alone sufficient to promote significant—albeit incomplete—silencing suppression by TCV P38.

## RESULTS

### VSR interactions with siRNA duplexes inhibit the loading, but not the activity, of AGO1/2-RISC

**FIGURE 1.**
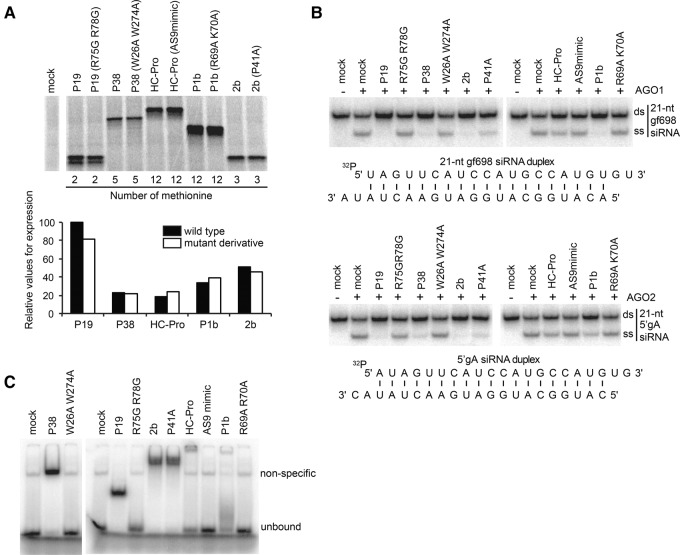
VSRs exhibit inhibitory effects on RISC loading. (*A*) Expression of TBSV P19, TCV P38, PVY HC-Pro, CVYV P1b, and CMV 2b, or the derivatives in BYL. In vitro translation reaction was performed using BYL in the presence of ^35^S-labeled methionine. *Lower* panel indicates the relative expression levels of individual wild-type VSRs (filled bar) and the mutant derivatives (open bar). Values were calculated by dividing the signal intensity of translation product by the number of methionine (indicated *below* the upper panel) contained in each product. (*B*) Effect of VSRs or the derivatives on AGO1/AGO2-RISC loading. The 21-nt gf698 or 5′gA siRNA duplexes containing 5′ ^32^P-labeled guide strands (strand with 5′ U in gf698 duplex or that with 5′ A in 5′gA duplex) were incubated in BYL expressing AGO1 or AGO2 in the presence of individual VSRs or the derivatives indicated *above* the panel. (*C*) Gel mobility shift of ^32^P-labeled 21-nt gf698 siRNA duplexes induced by individual VSRs or their derivatives expressed in BYL.

The present study was initiated as a parallel investigation of the molecular modes of action of TBSV P19, TCV P38, *Cucumber mosaic virus* (CMV) 2b, PVY HC-Pro, and *Cucumber vein yellowing virus* (CVYV) P1b. These factors have all been characterized as VSRs in planta, and amino acids essential to their anti-silencing functions have been identified in all cases (Kasschau et al. 1997; Chu et al. 2000; Chen et al. 2008; Azevedo et al. 2010; Valli et al. 2011). The BYL system was applied for the de novo expression of wild-type VSRs and their corresponding point-mutant derivatives. Although the overall translation efficiency varied among VSRs, accumulation of the mutant derivatives was comparable to that of the wild-type protein in each investigated case, as assessed by incorporation of ^35^S-methionine (Fig. 1A).

The RISC loading reaction is recapitulated in BYL using *Nicotiana tabacum* AGO1 expressed by in vitro translation in combination with synthetic small RNA duplexes (Iki et al. 2010). The siRNA passenger-strand removal and degradation during RISC loading produces a diagnostic, single-stranded (ss) siRNA guide-strand that is stabilized upon its incorporation into AGO1. Thus, RISC loading was measured by quantifying accumulation of ^32^P-labeled 21-nt ss siRNA. The addition of wild-type VSRs to the reaction mixtures effectively inhibited AGO1-RISC loading, and each stable point-mutant derivative showed a clear impairment in the inhibitory effects (Fig. 1B, upper panel); among the VSRs tested, PVY HC-Pro had the weakest effect, which could be explained by its lowest translation rate in the lysate (Fig. 1A). In addition to AGO1, AGO2 has been assigned key antiviral roles and so we also tested impairment of AGO2-RISC loading by VSRs through expression of *Arabidopsis* AGO2 by in vitro translation. This study showed that all VSRs impaired AGO2-RISC loading (Fig. 1B, lower panel). Furthermore, gel mobility-shift of ^32^P-labeled siRNA duplexes was induced by all VSRs, but not their mutant derivatives (with a weaker effect for HC-Pro, consistent with its lower translation rate, as seen in Fig. 1A), except for 2b^P41A^ (Fig. 1C). The replacement of the proline residue, P41, to alanine (A) in *Tomato aspermy virus* 2b was shown to significantly decrease (∼10-fold) its affinity to 21-nt siRNA duplexes (Chen et al. 2008). Such difference in affinity might not be reflected in the gel mobility-shift assay or, alternatively, the corresponding substitution in CMV 2b might have a weaker effect on siRNA-duplex interaction (Fig. 1C) while still efficiently attenuating RISC loading (Fig. 1B). To test whether any of the VSRs impacted RISC activity downstream from its loading, AGO1-expressing BYL was first allowed to load 21-nt siRNAs, and the reaction was then mixed with VSR-expressing BYL. None of the VSRs tested impaired the ability of AGO1 to slice a ^32^P-labeled, long ssRNA substrate at the expected siRNA-complementary position (Supplemental Fig. S1); similar results were obtained with AGO2. These results confirm and expand previous evidence provided by earlier studies of *Tobacco etch potyvirus* HC-Pro, *Beet yellow virus* P21, and tombusvirus P19 (Lakatos et al. 2006; Schuck et al. 2013). We conclude that interactions with siRNA duplexes strongly correlate with the ability of P19, P38, HC-Pro, P1b, and 2b to inhibit the loading of AGO1/AGO2-programmed RISCs. However, none of these proteins prevents the downstream capacity of loaded AGOs to slice a cognate target RNA in vitro.

### TCV P38 inhibits the processing of artificial dsRNA into siRNAs in a saturable manner

**FIGURE 2.**
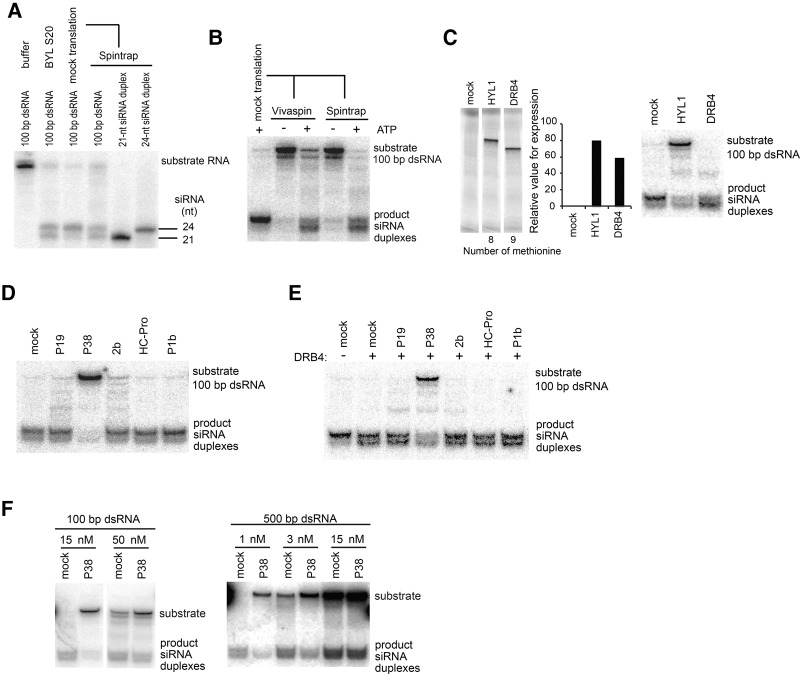
dsRNA processing activities are targeted by TCV P38. (*A*) Lengths of siRNAs generated by the processing activities in BYL. The RNA samples were analyzed in denaturing polyacrylamide gel electrophoresis to better distinguish the length difference of small RNAs than the nondenaturing conditions used regularly in the other experiments of this study. As size references, synthetic 21- and 24-nt siRNA duplexes containing ^32^P-labeled guide strand were incubated in the reaction mixtures and then extracted as for a 100-bp substrate dsRNA. (*B*) Efficient dsRNA-processing activities require ATP. After removing small molecules from the in vitro translation mixtures by ultrafiltration using Vivaspin, or by column purification with PD Spintrap G-25, dsRNA was incubated with or without the ATP-regeneration system. The ultrafiltration or column purification was not performed in the following dsRNA processing assays (*C*–*F*) because of a technical hindrance (see main text). (*C*) Expression of *A. thaliana* HYL1 and DRB4 by in vitro translation in the presence of ^35^S-labeled methionine, and the effect of HYL1 or DRB4 addition on dsRNA processing activities in BYL. (*D*) Effect of VSRs on dsRNA processing activities in BYL. (*E*) Effect of VSRs on dsRNA processing activities in DRB4-supplemented BYL. BYL expressing DRB4 and individual VSRs were mixed (1:1 v/v) before the incubation with dsRNA. (*F*) Effect of different lengths and dsRNA substrate concentrations on P38-mediated inhibition of dsRNA processing into siRNAs. The length and concentration of incubated dsRNA are indicated *above* the panel.

Antiviral PTGS is initiated and amplified through the processing of dsRNA into 21-nt and 22-nt siRNA duplexes by the activities of DCL4 and DCL2, respectively; competing for substrates in certain situations with DCL4 and DCL2, DCL3 produces 24-nt siRNA duplexes normally required for RdDM at the chromatin level (for review, see Pumplin and Voinnet 2013). To analyze the direct impact of VSRs on siRNA production, the endogenous dsRNA-processing activities residing in BYL were first investigated and characterized. When an experimental, ^32^P-labeled 100-base pair (bp) dsRNA was incubated in BYL, two siRNA species (upper band; 24-nt siRNA, lower band; 21-nt siRNA) were almost exclusively generated (Fig. 2A). However, the reaction mixture for in vitro translation consistently enhanced the accumulation of 24-nt siRNAs to the detriment of 21-nt species (Fig. 2A). This caveat prompted us to recondition the reaction mixtures by removing small molecules though column purification after the in vitro translation step. As a result of this treatment, the ^32^P-labeled dsRNA was processed into equally abundant 24-nt and 21-nt siRNA duplexes in a manner that was optimal under ATP-regenerating conditions in the BYL (Fig. 2B).

The activities of DCL proteins are assisted by dsRNA binding proteins, as exemplified by HYL1 for DCL1, and DRB4 for DCL4 (Dong et al. 2008; Fukudome et al. 2011). We thus examined the effects of exogenous addition of HYL1 or DRB4 on the dsRNA processing activities in BYL. For this purpose, *A. thaliana* HYL1 and DRB4 were expressed by in vitro translation to approximately similar levels and added to the dsRNA in vitro processing assay described above (Fig. 2C). HYL1 addition to the reaction mixture attenuated the processing of both 21- and 24-nt siRNA duplexes, with a stronger effect on the 24-nt species (Fig. 2C). In contrast, DRB4 addition facilitated the processing of 21-nt siRNA duplexes to the detriment of 24-nt species (Fig. 2C); similar results were obtained with a longer, 500-bp dsRNA (Supplemental Fig. S2A). These results support the idea that exogenously supplied HYL1 antagonizes BYL-maintained dsRNA processing activities relying on endogenous DCL3 and DCL4, while exogenous DRB4 assists the DCL4-mediated activity specifically. Similar dsRNA-processing properties have been established previously in *Arabidopsis* cell extracts (Nagano et al. 2014).

To examine the effect of VSRs on dsRNA processing into siRNAs, a ^32^P-labeled, 100-bp dsRNA was incubated in the reaction mixtures expressing VSRs by in vitro translation. Among all the VSRs tested, only P38 was able to efficiently inhibit dsRNA processing into 21- and 24-nt siRNAs, although a slight impingement to the reaction could be detected with P19 and 2b, accumulating longer processing intermediates (Fig. 2D); similar results were obtained when a 500-bp dsRNA was used as substrate (Supplemental Fig. S2B). One technical hindrance was that the column purification after in vitro translation (Fig. 2A,B) significantly attenuated the anti-dsRNA processing activity of TCV P38 (not shown). Thus, the effect of VSRs on dsRNA processing was investigated in lysate without column purification, a condition in which DCL3-dependent 24-nt siRNAs, involved in TGS, are predominantly produced (Fig. 2D). To better examine the effect of VSRs on the processing of PTGS-related, DCL4-dependent 21-nt siRNAs, the BYL was supplemented with DRB4, which enhances DCL4 activity and 21-nt siRNA production (Fig. 2C). Consistent with the results of Figure 2D, P38 inhibited dsRNA processing in BYL overexpressing DRB4, reducing accumulation of both 21- and 24-nt siRNAs (Fig. 2E). Of note, the inhibitory effects of P38 were reduced if it was mixed 1:1 with in vitro translated DRB4 (Fig. 2E), an effect likely due to the dilution of P38 compared to the conditions used in Figure 2D. The inhibitory effects of P38 were also strongly reduced if the amounts of 100-bp or 500-bp dsRNA substrate were progressively increased (Fig. 2F), indicating that P38 inhibits dsRNA processing in a manner that can be saturated by excess of substrate.

### TCV P38 inhibits processing of dsRNA produced by RDRs de novo

**FIGURE 3.**
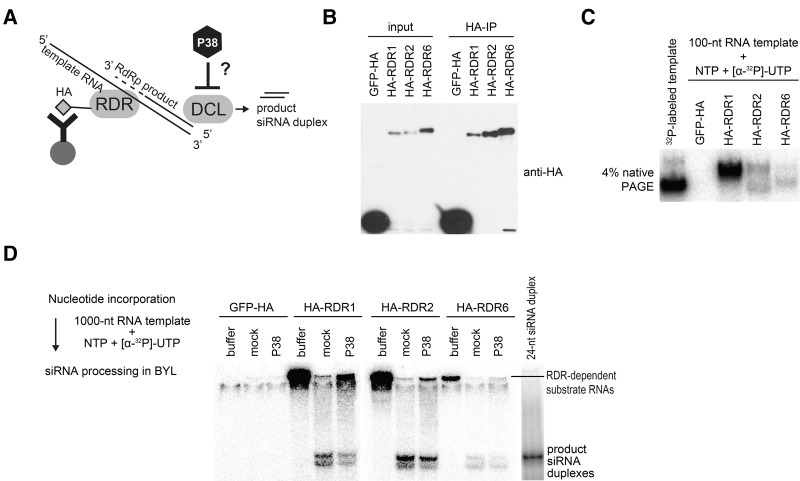
TCV P38 inhibits dsRNA processing from dsRNA synthesized by RDR proteins. (*A*) Schematic of the experiments conducted in *B–D*. (*B*) Immunoblot analysis of HA-tagged proteins. HA-RDR1, HA-RDR2, HA-RDR6, or GFP-HA (control) was expressed in BYL by in vitro translation (input) and immunopurified using anti-HA magnet beads (HA-IP). (*C*) Incorporation of nucleotides by immunopurified HA-RDR proteins. Nucleotide incorporation was performed using 750 nM 100-nt ssRNA as template in the presence of nucleotide mixture containing [α-^32^P]-UTP. In parallel, the same, but ^32^P-labeled template was incubated with HA-RDR6-enriched beads in the absence of [α-^32^P]-UTP. Following the reaction, RNA was extracted and analyzed by 4% native PAGE. (*D*) TCV P38 inhibits dsRNA processing activities on RDR-dependent RNA products. After the incubation for nucleotide incorporation, 2 µL of solution containing magnetic beads was mixed directly with 7 µL of TR buffer or BYL (undergoing mock or TCV P38 translation) together with 1 µL of ATP-regeneration mixture, and incubated at 25°C for 30 min.

An important step in antiviral RNA silencing is its amplification through the action of host-encoded RDRs, including chiefly RDR1, RDR6, and RDR2 (Garcia-Ruiz et al. 2010; Wang et al. 2010; Csorba et al. 2015). These enzymes use some viral ssRNA as templates to synthesize de novo complementary RNA strands, although the molecular characteristics of viral RDR substrates and their dsRNA products remain essentially unknown and may thus differ from the exogenous, in vitro synthesized, long dsRNA used in the BYL. We asked, therefore, if P38 can recognize dsRNA synthesized naturally by RDR proteins, as opposed to experimental dsRNA, and if this recognition impedes DCL-mediated dsRNA processing of RDR products. The BYL did not display any clear RDR activities, even after the expression of *Arabidopsis* RDR1, RDR2, or RDR6 by in vitro translation (data not shown). Based on a previous study showing that immunopurified *Arabidopsis* RDR6 expressed in *N. benthamiana* exhibits RDR activity (Curaba and Chen 2008), we resorted to using HA-epitope tagged RDRs expressed in BYL and subsequently immunopurified with anti-HA antibodies; HA-tagged GFP, used as a negative control for RDR activities, was immunopurified in parallel (Fig. 3A,B; Supplemental Fig. S3A). The HA-RDRs were then incubated with a cold 100-nt ssRNA in the presence of [α-^32^P]-UTP, to detect RDR activities by the incorporation of ^32^P (Fig. 3A,C). The same ^32^P-labeled 100-nt ssRNA template was run in parallel to provide a reference (Fig. 3C). In the presence of HA-RDR1, a high intensity band showing slower electrophoretic mobility than the reference ssRNA was observed in native PAGE analysis; no signal was detected upon incubation with the immunopurified HA-tagged GFP (Fig. 3C). A similar slower mobility band appeared in the presence of HA-RDR2, together with a second band migrating approximately at the size of the reference ssRNA; the weakest nucleotide incorporation was observed with HA-RDR6 (Fig. 3C), contrasting with its efficient immunopurification among the three RDRs tested (Fig. 3B). De novo ^32^P-labeling was also observed under native conditions upon incubation of a longer, 1000-nt template RNA with HA-RDR1, HA-RDR2, and HA-RDR6, but not with HA-tagged GFP (Fig. 3D, buffer conditions). It is noteworthy that RDR-dependent activities from either long or short templates were not associated with detectable dsRNA processing (Fig. 3D, buffer conditions). Therefore, the de novo ^32^P-labeled RNA was then directly incubated with BYL, where it should naturally promote endogenous DCL4 and DCL3 activities, generating 21- and 24-nt siRNAs, respectively (Fig. 2A,B). The incubation indeed led to the production of cognate siRNA species (Fig. 3D), confirming the double-stranded nature of the de novo ^32^P-labeled RNA generated by the immunoprecipitated RDRs. In all three cases, siRNA production was impaired by the addition of TCV P38 in BYL by in vitro translation, coinciding with the over-accumulation of longer dsRNA precursors (Fig. 3D). Therefore, TCV P38 inhibits the processing of not only experimental in vitro transcribed dsRNA added to the lysate, but also of cognate RDR-dependent products, which presumably mimic those synthesized during the amplification stages of antiviral PTGS and that may display distinct biochemical features from experimental dsRNA. We note that the above setting could also be used in future experiments to assess the specific effects of various VSRs on the RDR-mediated de novo dsRNA synthesis step.

### Inhibition of RISC loading and dsRNA processing are recently acquired properties of TCV P38

**FIGURE 4.**
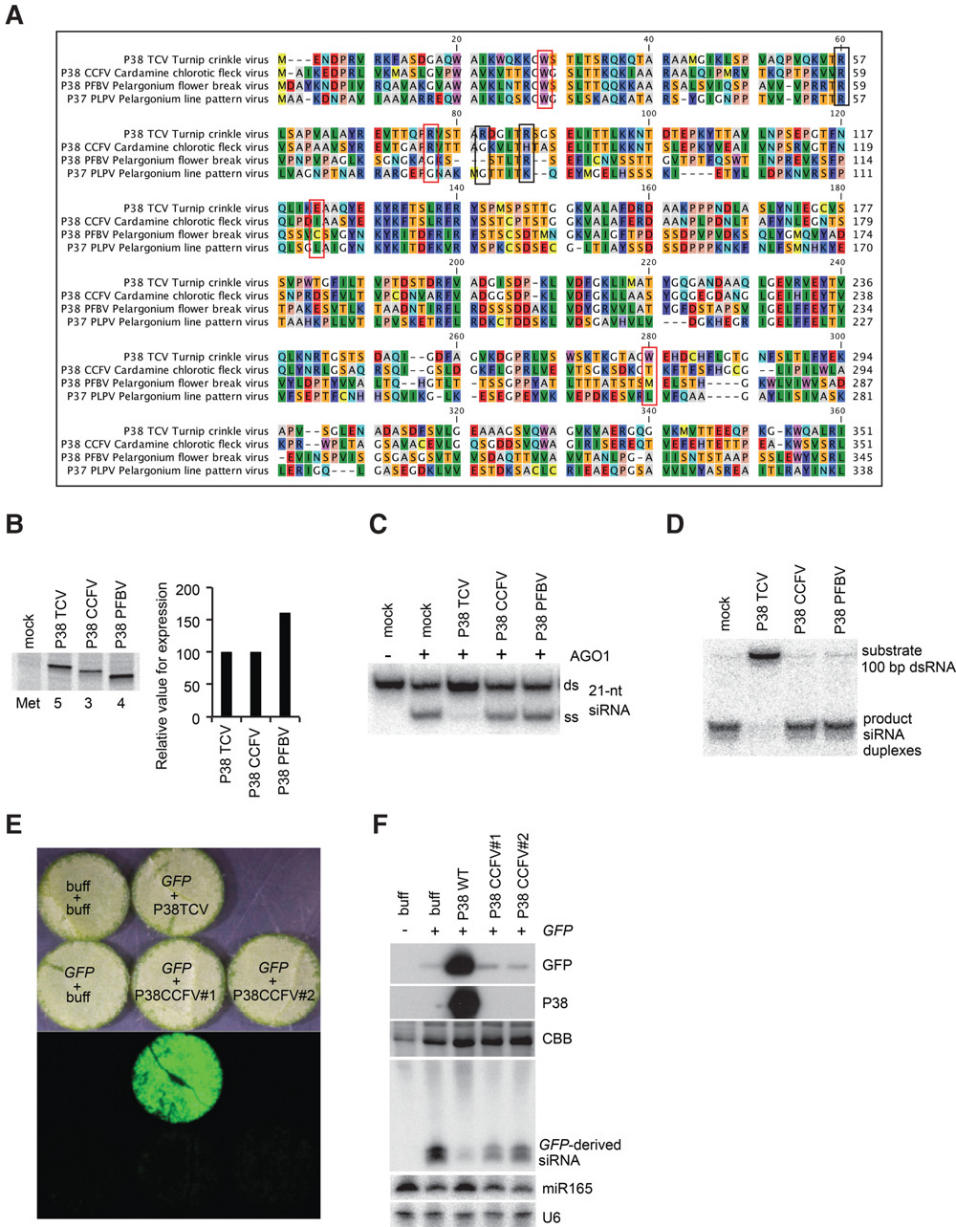
Comparative analysis on Carmovirus P38 proteins. (*A*) Alignment of Carmovirus P38 proteins. TCV P38 (ADT78694), CCFV P38 (NP_041887.1), PFBV P38 (ABD93258.1), and PLPV P37 (ACJ38486.1) were aligned using CLC Genomics Workbench 8.0.2. Amino acid residues of TCV P38 analyzed in this study are marked by squares. Red squares highlight the residues characterized in the former studies (Deleris et al. 2006; Azevedo et al. 2010). (*B*) Relative expression levels of P38 proteins of TCV, CCFV, and PFBV, in BYL. In vitro translation reaction was performed in the presence of ^35^S-labeled methionine, and the expression values were calculated as described in Figure 1A. (*C*) Effect of P38 proteins on AGO1-RISC loading in BYL. (*D*) Effect of P38 proteins on dsRNA processing in BYL. (*E*,*F*) Effect of TCV or CCFV P38 on the expression of GFP in *N. benthamiana* leaves. GFP fluorescence signals (*E*) and the accumulation of proteins or the GFP-derived RNA fragments (*F*) were analyzed in parallel. The Coomassie brilliant blue (CBB) staining or the accumulation of miR165 and U6 serve as loading control for Western and Northern analyses, respectively.

Based on the inhibitory effects of TCV P38 on dsRNA processing and RISC loading, we tested whether P38 proteins from other carmoviruses share similar attributes. The P38 proteins encoded by *Cardamine chlorotic fleck virus* (CCFV) and *Pelargonium flower break virus* (PFBV) (Fig. 4A) were thus expressed by in vitro translation, alongside TCV P38, to comparable levels (Fig. 4B). Unlike TCV P38, however, neither CCFV P38 nor PFBV P38 showed any effect on RISC loading or dsRNA processing (Fig. 4C,D). Recently, *Pelargonium line pattern virus* (PLPV) P37 (a protein related to TCV P38, Fig. 4A) was shown to act as a VSR and to bind siRNA duplexes but not long dsRNA in *N. benthamiana* leaf extracts (Perez-Canamas and Hernandez 2015). Phylogenetic analysis of carmovirus P38 proteins indicates that TCV P38 is more similar to CCFV P38 than it is to PLPV P37 (Supplemental Fig. S4). We thus analyzed the CCFV P38 in a standard transient silencing suppression assay conducted in leaves of wild-type *N. benthamiana*, in which the GFP mRNA is both an inducer and a target of RDR6-dependent sense-PTGS. Coexpression of a VSR prevents GFP silencing, leading to enhanced green fluorescence under UV illumination and high GFP protein accumulation. However, the coexpressed VSR mRNA is also an intrinsic target of sense-PTGS in this assay, such that weak or loss-of-function VSR alleles usually accumulate poorly, if at all, in the infiltrated leaf. As seen on Figure 4E and F, the GFP protein levels were strongly enhanced in samples cotreated with TCV P38, compared to those cotreated with buffer or with CCFV P38, confirming its lack of VSR activity compared to TCV P38.

Together, these observations strongly suggest that the inhibitory activities of TCV P38 against RISC loading and dsRNA processing, as well as the siRNA binding activity of PLPV P37, represent recent additions to the core capsid function conserved among all *Carmoviridae* as demonstrated experimentally (e.g., P38, P37) or inferred from structural modeling based on the *T* = 3 icosahedral capsid of TCV, other carmoviruses, and the *Tombusviridae* (Harrison et al. 1978; Hogle et al. 1986; Azevedo et al. 2010; Bakker et al. 2012).

### siRNA binding and RISC-loading inhibition are essential and tightly coupled steps of TCV P38 VSR function

An evolutionarily conserved platform to bind AGOs consists of one of several pairs of tryptophan/glycine (WG) or GW residues (Karlowski et al. 2010). A previous report showed that the two discrete GW motifs residing at W27 and W274 of TCV P38 (Fig. 4A, red squares) are required for its association to AGO1 in vitro and its VSR function in vivo (Azevedo et al. 2010). Additionally, two missense yet stable mutations in arginine (R74) and glutamate (E122) residues of TCV P38 (Fig. 4A, red squares) were also shown to cause loss-of-P38-mediated suppression of silencing; structurally, R74 and E122 are in close proximity at the interface facilitating C:C homo-dimerization of P38, required for its VSR activity (Deleris et al. 2006; Azevedo et al. 2010).

**FIGURE 5.**
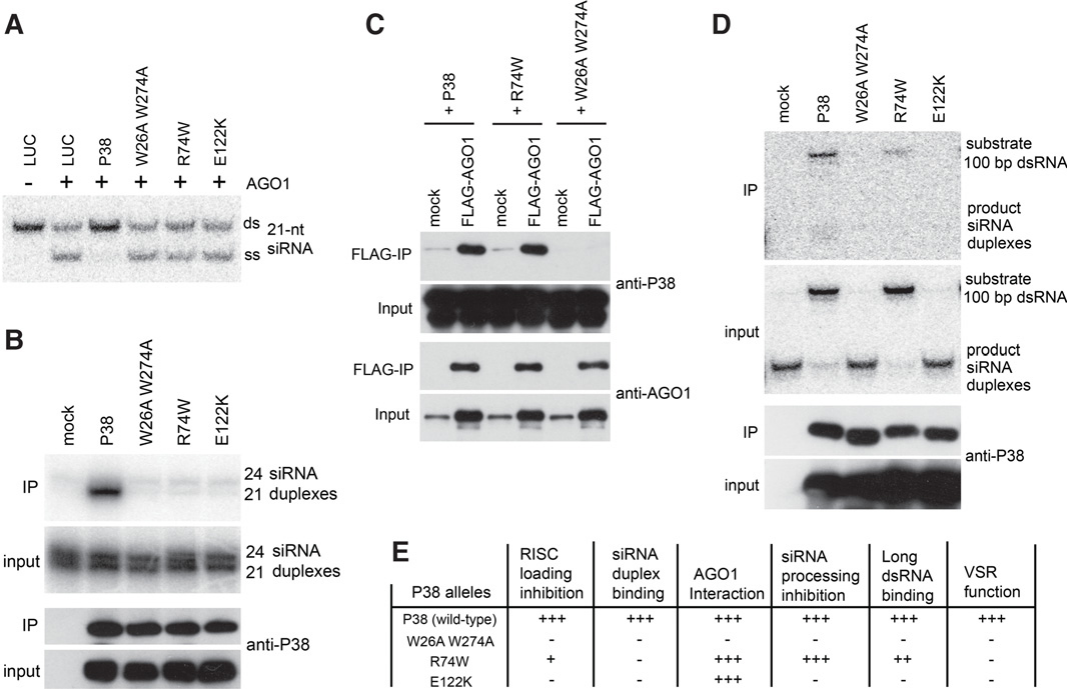
Side-by-side analysis of TCV P38 and mutant derivatives in BYL. (*A*) Effect of TCV P38 and derivatives on AGO1-RISC loading. Luciferase (LUC) was used as control. (*B*) Size-selective copurification of siRNA duplexes with TCV P38. In vitro translation mixtures were incubated with ^32^P-labeled 24-nt and 21-nt siRNA duplexes (10 nM each) for 60 min, followed by immunopurification using anti-P38 antibody. LUC was used as control. (*C*) Physical interaction between AGO1 and TCV P38 or its mutant derivatives. BYL expressing Flag-AGO1 (mock translation as control) and individual P38 proteins were mixed (1:1 v/v) before immunopurification using anti-Flag antibody. (*D*) Effect of TCV P38 or the derivatives on dsRNA processing, and interaction of TCV P38 or the derivatives with dsRNA in dsRNA-processing reaction. In vitro translation mixtures were incubated with ^32^P-labeled dsRNA, followed by immunopurification using anti-P38 antibody. (*E*) Summary of the activities of TCV P38 and its mutant derivatives.

We thus tested the effects of these previously identified nonfunctional P38 alleles in BYL. Upon introduction of the cognate point mutation(s), the mRNAs of the *P38* alleles were in vitro translated and the effects of the corresponding proteins (P38^W26A W274A^, P38^R74W^, and P38^E122K^) on RISC loading and siRNA interaction were analyzed in parallel. Having confirmed the synthesis of near-identical amounts of wild-type and mutant P38 alleles, we found that P38-mediated RISC-loading inhibition was strongly attenuated by each of the amino acid substitutions tested (Fig. 5A). Consistent with the gel mobility shift assay (Fig. 1C), immunoprecipitation analyses showed that siRNA duplexes were copurified with P38, but not with P38^W26A W274A^ (Fig. 5B). Similar to P38^W26A W274A^, neither P38^R74W^ nor P38^E122K^ displayed detectable affinity for siRNA duplexes (Fig. 5B). Of note, P38–siRNA duplex interaction was strongly size-selective for 21-nt siRNA duplexes when both 21- and 24-nt siRNA duplexes accumulated at similar levels. This result argues against the previously demonstrated size-independent interaction of P38 with dsRNA in *N. benthamiana* leaf extract (Mérai et al. 2006). We next examined the physical interaction between P38 and AGO1 in BYL. While P38, P38^R74W^, and P38^E122K^ copurified with Flag-tagged AGO1 expressed by in vitro translation (Fig. 5C; Supplemental Fig. S5), this was not the case with P38^W26A W274A^, as previously reported in vitro and in vivo (Azevedo et al. 2010). Taken together, these results indicate the importance of P38–siRNA interactions for RISC-loading inhibition, and that the previously characterized physical interaction of P38 with AGO1 (Azevedo et al. 2010), while likely necessary, is not sufficient to exert RISC-loading inhibition.

The effects of P38 derivatives (P38^W26A W274A^, P38^R74W^, and P38^E122K^) on the processing of long dsRNA were then examined. P38^W26A W274A^ and P38^E122K^ were both impaired in dsRNA-processing inhibition, while P38^R74W^ retained this ability at a level comparable to that of wild-type P38 (Fig. 5D, input). Immunopurification using P38 antibody showed that ^32^P-labeled dsRNA substrates could copurify with both P38 and P38^R74W^, albeit slightly less efficiently with the latter (Fig. 5D, IP). This result with P38^R74W^ suggests that interaction with long dsRNA combined with dsRNA-processing inhibition can be uncoupled from P38–siRNA interaction and RISC-loading inhibition. They also reinforce the notion that the two latter processes are tightly connected and essential for the P38 VSR function (see Fig. 5E for a summary of the effects of each mutation).

### Discrete point mutations in TCV P38 uncouple impairment of RISC-loading from dsRNA-processing inhibition

**FIGURE 6.**
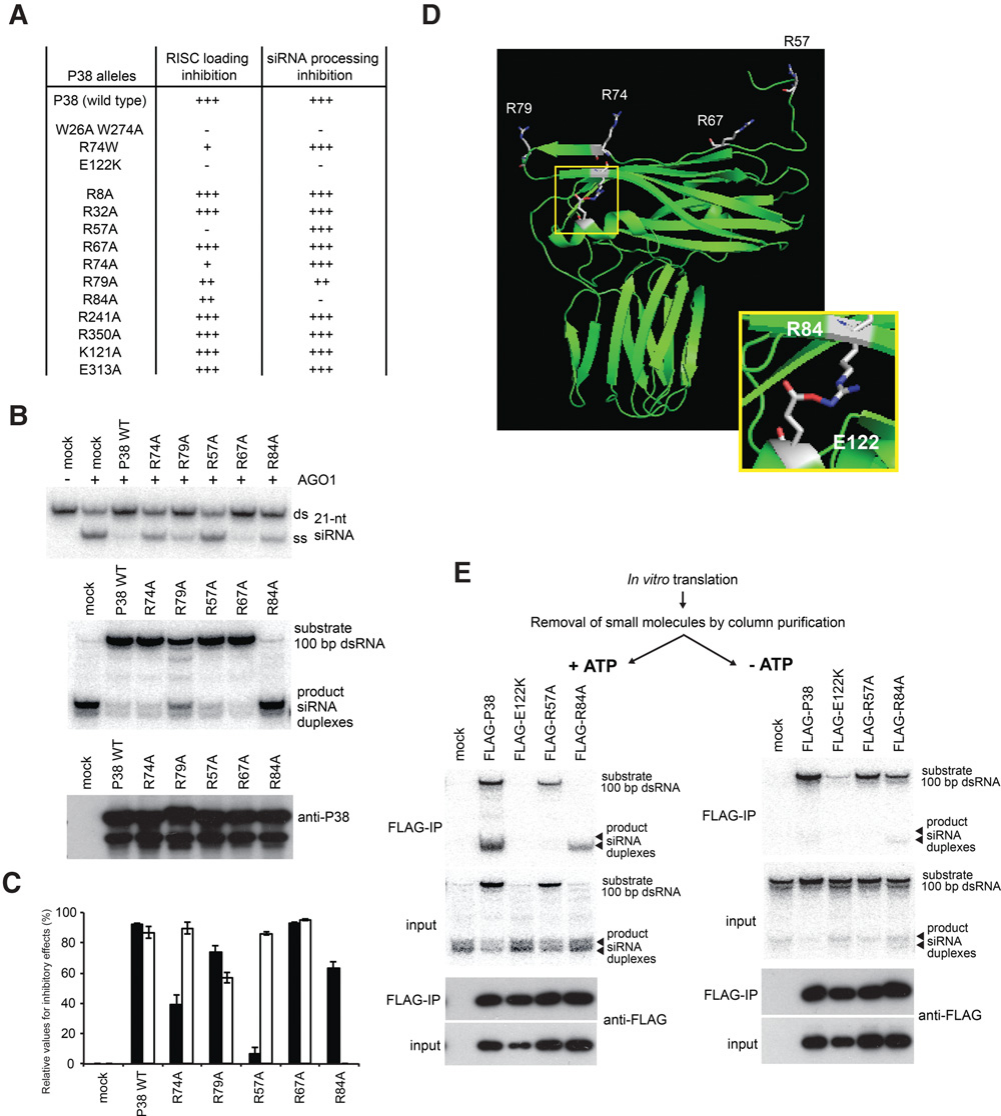
Alanine-scanning mutagenesis of TCV P38. (*A*) Summary of serial alanine scanning screening. (*B*) Effect of generated P38 derivatives on AGO1-RISC loading (*top*) and dsRNA processing (*middle*). The *bottom* panel shows the immunoblotting for the P38 proteins expressed in BYL. (*C*) Relative values for the inhibitory activities induced by P38 and mutant derivatives against RISC loading (filled bar) and dsRNA processing (open bar). The 100% inhibition defines the conditions when siRNA duplex was not single-stranded in RISC loading (as in the absence of AGO1 expression) or when no siRNA products were detected upon dsRNA processing. (*D*) Structural information on TCV P38. The analyzed amino acid residues in Figure 4A are highlighted. The 84th arginine and 122th glutamate residues form a salt bridge. The panel was created by using PyMol with the structural data on TCV P38 (PDB: 3ZX8). (*E*) Copurification of dsRNA substrate and the product siRNA duplexes with P38 and derivatives. Small molecules were removed from the reaction mixtures after in vitro translation, and then the incubation with dsRNA was performed with (+ATP) or without (−ATP) the addition of ATP-regenerating system, followed by immunopurification using anti-Flag antibody.

To gain further insight into the RISC-loading and dsRNA-processing inhibitory functions of TCV P38, alanine-scanning mutagenesis was performed (Fig. 6A) mainly on arginine residues, given that the positively charged moieties have the potential to mediate RNA–protein interactions, as exemplified with the substitution of R74 to W, required for sRNA-duplex binding and RISC-loading inhibition (Fig. 5A,B,E). The screen (Fig. 6A) identified several stable alleles of P38, which were expressed at levels similar to those of the wild-type protein, and of which some displayed noticeable properties (Fig. 6B,C). Hence, the R57A substitution was specifically impaired in RISC-loading inhibition but retained a strong ability to suppress dsRNA processing into siRNAs (Fig. 6B,C). A similar phenotype was observed for P38^R74W^ or its P38^R74A^ derivative, although the R57A substitution impaired RISC-loading inhibition more severely than the R74W/A substitution (Figs. 5A or 6B,C, respectively). The screen also identified P38^R79A^, which, in contrast to the P38^R57A^ and P38^R74W/A^ alleles, partially lacks inhibitory activities against both RISC loading and dsRNA processing (Fig. 6B,C). Finally, we isolated P38^R84A^, which displays a near-complete impairment in dsRNA-processing inhibition while maintaining significant suppression of RISC loading (Fig. 6B,C). However, the severe impairment caused by substituting R84 should be considered in the context of potential conformational changes imposed upon TCV P38, given that R84 forms a salt bridge with E122 (Fig. 6D), a residue genetically identified as essential for P38 VSR activity most likely due to its position at the interface of C:C homodimers (Deleris et al. 2006; Azevedo et al. 2010). We note that E122 is not conserved in the nonfunctional P38 alleles from CCFV and PFBV (Fig. 4A), reinforcing the notion that silencing suppression is a likely newly acquired property of the TCV-encoded P38. Gel mobility-shift assay and immunopurification precipitation experiments confirmed the tight correlation between the interaction of TCV P38 with siRNA duplexes and its inhibitory activity against RISC loading (Fig. 6A,B; Supplemental Fig. S6A,B).

### dsRNA binding by P38 is required for the inhibition of processing into siRNAs

To examine further the interaction between the various TCV P38 alleles and the dsRNA processing substrates, immunopurification of Flag-tagged versions of TCV P38 using anti-Flag antibody was conducted. The N-terminal Flag tag on TCV P38 did not affect its inhibitory activities against RISC loading and dsRNA processing (data not shown, and Fig. 6E). In addition, immunopurification using Flag tag enriched P38 proteins more efficiently than by using the anti-P38 antibody (data not shown). As a result, both substrate dsRNA and product siRNA duplexes (mainly 21-nt species) could be copurified with Flag-P38 (Fig. 6E). A substantial amount of substrate dsRNA was copurified with Flag-P38^R57A^, albeit less efficiently than with Flag-P38. Consistent with the fact that the substitution on R57 impairs the interaction of TCV P38 with synthetic siRNA duplex (Supplemental Fig. S6A,B), negligible copurification of product siRNA duplexes was detected with Flag-P38^R57A^ (Fig. 6E). As noted in Figure 2, the column purification after in vitro translation reaction had attenuated the inhibitory effect of P38 on dsRNA processing. However, the affinity of P38^E122K^ and P38^R84A^ to substrate dsRNA could not be examined in the ATP-available condition, in which dsRNA is processed very efficiently. To circumvent this caveat, immunopurification was performed in ATP-depleted, thus dsRNA processing-inefficient, conditions in which most of the substrate dsRNA remained intact (Fig. 2B). As seen under ATP-available conditions, dsRNA was copurified with Flag-P38^R57A^ at a level comparable with Flag-P38 under ATP-depleted conditions (Fig. 6E). In contrast, the amount of copurified dsRNA was moderately and severely reduced by substitutions on R84 and E122, respectively (Fig. 6E). These results indicate that the interaction of TCV P38 with substrate dsRNA is linked to its inhibitory activity against dsRNA processing into siRNAs.

### In vivo experiments corroborate the existence of genetically separable, dual activities by TCV P38

**FIGURE 7.**
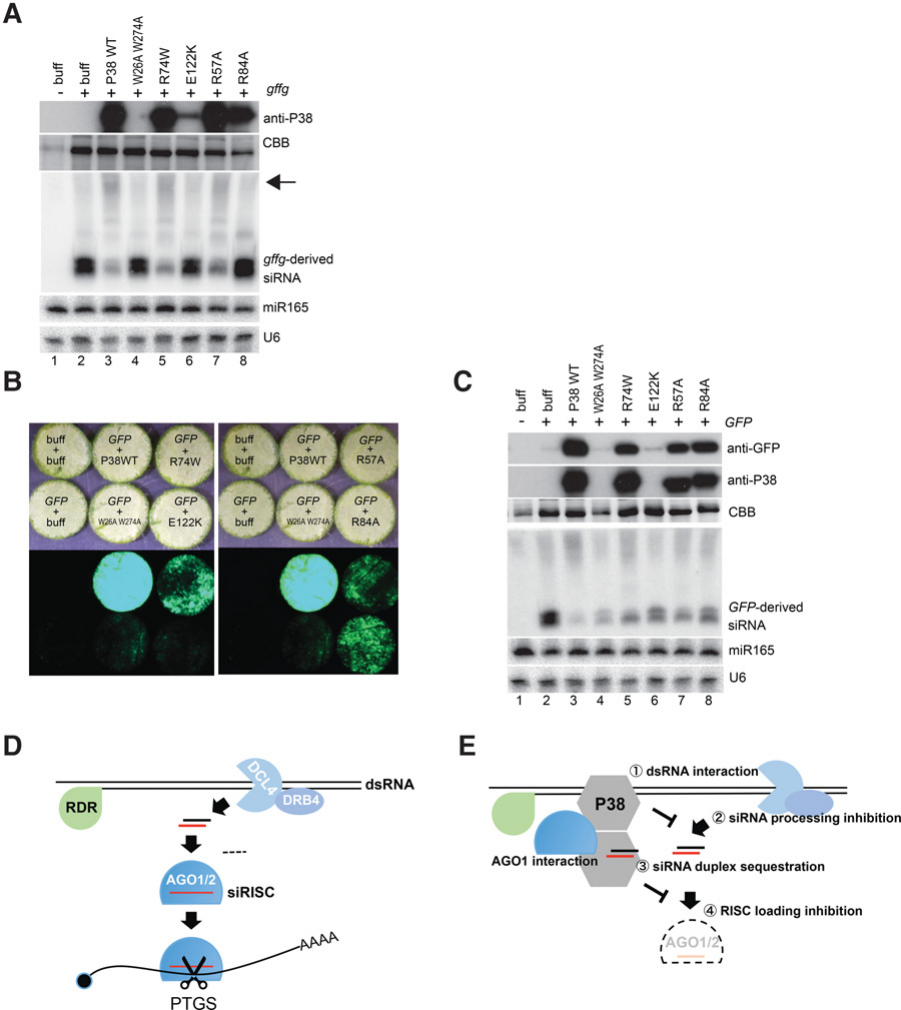
Expression of TCV P38 or the mutant alleles in vivo. (*A*) Effect of TCV P38 or the mutant alleles on the accumulation of inverted repeat (*gffg*)-derived siRNAs in *N. benthamiana* leaves. The Coomassie brilliant blue (CBB) staining, or the accumulation of miR165 and U6 serve as loading control for Western and Northern analyses, respectively. The arrow indicates the higher molecular weight materials accumulated upon *gffg* expression. (*B*,*C*) Effect of TCV P38 or the mutant alleles on the expression of GFP in *N. benthamiana* leaves. GFP fluorescence signals (*B*) and the accumulation of proteins or the GFP-derived RNA fragments (*C*) were analyzed in parallel. Among the six leaf discs shown in panel *B*, the *left* four leaf discs are identical. (*D*) Model for antiviral PTGS in plants. (*E*) Model for the attenuation of antiviral PTGS by TCV P38. The R57A or R74A/W alleles are specifically impaired for RISC-loading inhibition associated with siRNA-duplex sequestration, but retain the ability to interact with dsRNA and to inhibit the processing into siRNAs, accounting for partial suppression of PTGS. In contrast, the R84A allele is primarily impaired in dsRNA-processing inhibition, but retains the ability to sequester siRNA duplexes and to inhibit RISC loading, accounting for partial suppression of PTGS.

To further ascertain in vivo the various findings made with TCV P38 in vitro, key P38 alleles were subjected to transient silencing suppression assays in leaves of wild-type *N. benthamiana*. The first assay entails the combined expression of P38 or its mutant derivatives together with an RDR6-independent inducer of siRNA production consisting of an inverted-repeat formed with the 5′ sequence of the *GFP* gene, hereafter referred to as *gffg*. This experiment is conceptually similar to the in vitro setting presented in Figures 2, 4D, 5D, 6B (middle panel), and 6E. As expected, expression of *gffg* with buffer led to the production of 21- and 24-nt *gffg*-derived siRNAs, whose accumulation was strongly reduced upon coexpression of wild-type TCV P38 (Fig. 7A, tracks 2 and 3), consistent with the results of the in vitro dsRNA-processing and RISC-loading inhibition experiments (summarized in Figs. 5E, 6A). It is noteworthy that the TCV P38 effect was associated with accumulation of higher molecular weight material (Fig. 7A, arrow) that likely corresponded to unprocessed, long *gffg* dsRNA. In contrast, the W26A W274A and E122K alleles had little effect on siRNA accumulation, and accordingly, both were below detection limit in Western analysis, owing to the inability of these P38 alleles to protect their own mRNA against sense-PTGS, which is intrinsically induced by the transient expression method (Fig. 7A, tracks 4 and 6). These results are consistent with the complete inability of P38^W26A W274A^ and P38^E122K^ to inhibit both dsRNA processing and RISC loading in vitro (Fig. 6A). The R74W and R57A alleles accumulated in Western analysis and both suppressed *gffg*-derived siRNA accumulation, albeit to a lower extent than wild-type P38 (Fig. 7A, tracks 5 and 7, arrow). This partial effect is consistent with the unaltered capacity of both alleles to inhibit dsRNA processing into siRNAs in vitro (Fig. 6A) and in vivo (Fig. 7A), combined with their strongly reduced (R74W) or nonexistent (R57A) ability to inhibit RISC loading (Fig. 6A,B, upper panel, 6C). Hence, the residual *gffg*-derived siRNAs seen in the P38^R74W^- and P38^R57A^-treated samples most likely correspond to low amounts of processed siRNAs that remain protected from degradation in vivo due to their loading into AGO1/2, a step normally impeded by wild-type P38 (Fig. 7A, track 3). Finally, the R84A allele also accumulated in Western analysis but did not prevent *gffg*-derived siRNA accumulation (Fig. 7A, track 8), consistent with its complete inability to inhibit dsRNA processing (Fig. 6A, 6B, middle panel, 6C), but preserved the capacity to bind siRNAs (Fig. 6E, left panel) and to impair (∼60%) RISC loading in vitro (Fig. 6B, upper panel, 6C).

To further ascertain the above results, we used a second silencing suppression assay involving, as in Figure 4E and F, a transiently expressed *GFP* mRNA acting both as an inducer and a target of RDR6-dependent sense-PTGS. As in the previous experiment, the W26A W274A and E122K alleles were both below detection levels in Western analysis and failed to suppress *GFP* silencing, correlating with the accumulation of *GFP*-derived siRNAs (Fig. 7B, 7C, tracks 4 and 6). A similar siRNA-accumulation pattern was observed upon expression of P38^R84A^ (Fig. 7C, track 8). However, the R84A allele partially de-repressed GFP expression (Fig. 7B and 7C, track 8), possibly due to the ability of this allele to impair (∼60%) RISC loading in vitro (Fig. 6B, upper panel, 6C). Coexpression of the wild-type TCV P38 strongly enhanced GFP accumulation and reduced *GFP* siRNA accumulation, compared to those seen in buffer cotreated samples (Fig. 7B, 7C, tracks 2 and 3). Like that of *gffg*-derived siRNAs (Fig. 7A), the accumulation of 21-nt *GFP* siRNAs was enhanced during expression of P38^R74W^ or P38^R57A^, compared to those remaining during wild-type P38 expression (Fig. 7B, 7C, tracks 5 and 7 versus track 3), since these alleles efficiently prevent dsRNA processing into siRNAs in vitro (Fig. 6A, 6B, middle panel) and in vivo (Fig. 7A and 7B, arrows), but unlike the wild-type P38, they failed to inhibit AGO1/2 loading with siRNAs. Importantly, the results with the R74W and R57A alleles indicate that inhibition of dsRNA processing, which requires dsRNA binding (Fig. 6E), is alone sufficient to promote significant—albeit incomplete—silencing suppression by TCV P38 in vivo.

## DISCUSSION

In planta studies of VSRs produced in isolation from constitutively or transiently expressed transgenes, or from bona fide virus replication have been so far the prevalent, if not unique, mode of investigation of these proteins. However, due to the in vivo complexity and intertwined nature of silencing pathways, the outcome of in planta studies of VSRs do not always provide sufficient evidence to describe their molecular activities. As demonstrated by this study, the cell-free analysis based upon plant lysates allows the systematic investigation of potentially each individual step of the RNA silencing reaction, in isolation of the others, enabling a highly reductionist approach of the process, thus providing a valuable complement to in vivo studies. So far, plant lysates have been mostly used to decipher the RNA silencing mechanism itself, but they have been rarely used in combination with VSRs, an approach we have now established in BYL by recapitulating each of the key RNAsilencing steps potentially involved in antiviral defense. While we found commonalities among the various VSRs tested in their ability to prevent the loading, but not the activity, of the siRNA-RISC, TCV P38 emerged as displaying the additional and unique ability to prevent processing of siRNA from long dsRNA produced either synthetically or from the activities of plant RDRs in the BYL. Suppression of the most upstream step of RNA silencing by P38 has been inferred from in planta studies (Deleris et al. 2006; Mérai et al. 2006), but never further investigated. Owing to the availability of stable mutant alleles of P38, use of the BYL has allowed us to dissect dsRNA processing inhibition and notably its relevance to the ability of TCV P38 to bind long dsRNA. This study leads us to propose a dual-activity model for TCV P38 action in which dsRNA processing inhibition, which requires P38 binding to long dsRNA is alone sufficient to promote significant silencing suppression in a manner genetically separable from the downstream suppression of RISC loading via AGO1/2 interaction and siRNA duplex sequestration. Hence, R84 is primarily required for efficient dsRNA interaction and dsRNA-processing inhibition, while R57 and R74 are specifically required for siRNA-duplex interaction and RISC-loading inhibition, indicating that siRNA-binding/RISC-loading inhibition and dsRNA-interaction/dsRNA-processing inhibition are dissociable, and thus likely independent activities of TCV P38 (Fig. 7E). Such discriminative action against long dsRNA and siRNA duplexes by a single protein is a unique property among the plant virus-encoded VSRs studied thus far and is also not evident in the B2 protein of invertebrate viruses such as *Flock House virus* (FHV) and *Wuhan nodavirus* (WhNV), which share several functional similarities with TCV P38, as uncovered here (Chao et al. 2005; Qi et al. 2011). Similarly, yet not discriminated, the dicistrovirus VSR, *Drosophila C virus* (DCV) 1A was shown to inhibit both Dcr2-mediated dsRNA processing and Ago2-RISC assembly (van Rij et al. 2006; Nayak et al. 2010). On the other hand, the closely related *Cricket paralysis virus* (CrPV) 1A does not share such dual functions of DCV-1A but acts as VSR by inhibiting Ago2-RISC-mediated slicing (Nayak et al. 2010).

One further aspect revealed in this study is that P38 alleles isolated from related carmoviruses were inert in both the BYL- and *Agrobacterium*-mediated silencing suppression assays, despite accumulating to similar levels as TCV P38 and the conservation of an N-terminal GW motif previously implicated in direct AGO binding (Azevedo et al. 2010). Given the conservation of the P38 open reading frame among carmoviruses, an interpretation is that silencing suppression is a sporadic and perhaps recent addition to a core structural function of P38 shared among all carmoviruses, which, we propose, is the encapsidation of virions. This uncoupling between virion formation and silencing suppression is in line with previous conclusions drawn from in vivo studies (Deleris et al. 2006). Alternatively, it is possible that some of the P38 proteins of the non-TCV viruses tested here do function as VSRs in other hosts than *Nicotiana* species from which the BYL is derived (*N. tabacum*) and in which the transient assay for silencing suppression in vivo was conducted (*N. benthamiana*). In any case, these results suggest that caution should be exerted in extrapolating VSR functions from proteins of related viruses.

Overall, this study demonstrates how the BYL could serve as a unifying biochemical platform for the discovery and comparison of the biological modes of action of VSRs encoded by different viral genera. Indeed, BYL can synthesize VSR mutant derivatives as efficiently as the wild-type proteins, enabling side-by-side analyses under comparable expression levels. Further technical refinement beyond the BYL-based RISC loading, dsRNA processing and binding assays should not only provide novel insights into the molecular activities of VSRs but also help uncover the VSR-targeted molecular mechanisms underlying (antiviral) RNA silencing pathways. Ultimately, however, findings made in vitro or upon transgenic VSR expression in isolation of the virus should be pondered in the context of authentic infections, which are likely to provide a much more complex and accurate view of VSR function and their effects in planta, as recently shown in unraveling studies of the Hc-Pro and P19 proteins during real infections (Garcia-Ruiz et al. 2015; Kontra et al. 2016).

## MATERIALS AND METHODS

### Plasmid construction for mRNA preparation

Fragments of TBSV *P19* (GenBank: AJ288942.1) and the derivative (R75G R78G), TCV *P38* (GenBank: HQ589261.1) and a derivative (W26A W274A), CMV *2b* (strain Y, GenBank: D12538.1) or *2b* (strain Q, GenBank: Z21863.1), PVY *Hc-Pro* (GenBank: AFR11765.1), and CVYV *P1b* (GenBank: DQ496114.1) were amplified by PCR. Fragments of *Cardamine chlorotic fleck virus* (CCFV) *P38* (NC_001600.1), *Pelargonium flower break virus* (PFBV) *P38* (GenBank: DQ443018.1) were synthesized by GeneArt Strings (Thermo Fisher Scientific). DNA fragments encoding *HYL1*, *DRB4*, *RDR1*, *RDR2*, or *RDR6* were amplified by PCR using cDNA prepared from RNA of *A. thaliana* Col-0. All the fragments were cloned into pSP64-poly(A) vector (Promega) using appropriate restriction sites. Substitution of nucleotides or addition of epitope sequences on genes was performed by PCR using plasmid clones as templates and primers containing relevant sequences. Oligonucleotides are listed in Supplemental Table S1. Each messenger RNA was prepared from the linearized plasmids using AmpliCap SP6 High Yield Message Maker Kit (Cellscript).

### In vitro translation

The preparation of BYL and in vitro translation reaction were as described previously (Komoda et al. 2004). Membranous fraction of BYL was pelleted by centrifugation (21,000*g*, 15 min), and the cytosolic fraction (S20) was used for in vitro translation in this study. All mRNAs were translated at 0.05 µg/µL in reaction mixtures. The expression was measured by the incorporation of ^35^S-labeled methionine in the translation reaction in the addition of ^35^S-labeled methionine to the amino acid mixtures excluding methionine (Promega). The reaction mixture was boiled in WB loading buffer (10%[v/v] glycerol, 4%[v/v] SDS, 62.5 mM Tris–HCl [pH 6.8], 5%[v/v] β-mercaptoethanol, and 0.0075% bromophenol blue), and analyzed in NuPAGE 4%–12% Novex Bis-Tris protein gels (BioRad). The signals were detected using BAS-MS imaging plate (FUJIFILM) and Typhoon FLA 9000 image analyzer (GE Healthcare). The relative expression level was calculated by dividing signal intensity of translation product by the number of methionine of each protein.

### Small RNAs

The sequence information for 21- and 24-nt gf698 siRNAs was described previously (Ye et al. 2012; Yoshikawa et al. 2013). All small RNAs had 2′-hydroxymethyl groups on the 3′-terminal nucleotides. The guide strands were phosphorylated in the presence of [γ-^32^P]-ATP by T4 polynucleotide kinase (Thermo Fisher Scientific), while the passenger strands were also phosphorylated but without radiolabeling. The annealing was performed during the incubation at 96°C for 2 min followed by a gradual temperature decrease in the annealing buffer composed of 10 mM Tris–HCl (pH 7.6), 20 mM KCl, and 1 mM MgCl_2_.

### Gel mobility shift analysis

After in vitro translation, the reaction mixtures were incubated with 20 nM ^32^P-labeled 21-nt siRNA duplexes at 25°C for 15 min, and then mixed with an equal amount of native dye solution, and analyzed in 5% native PAGE using 0.5× TBE as the running buffer (75 V for 40 min).

### RISC loading reaction

*Nicotiana tabacum* AGO1, VSRs, and the derivatives were expressed in BYL by in vitro translation, mixed (1:1 v/v) and incubated at 25°C for 60 min with 10 nM 21-nt gf698 siRNA duplexes containing 5′ ^32^P-labeled guide strand in the additional presence of ATP-regenerating system composed of 0.75 mM ATP, 1 mM MgCl_2_, 20 mg/mL creatine phosphate, and 0.4 mg/mL creatine kinase. For AGO2 RISC loading, *A. thaliana* AGO2 was used with 5′gA siRNA duplex containing guide strand with 5′ adenosine. To analyze RNA, the reaction mixtures were diluted 10-fold with 10 mM Tris, 1 mM EDTA (TE, pH 8.0) and extracted with equal volumes of phenol chloroform isoamyl alcohol (PCI, 25:24:1, v/v). The resulting aqueous phase was recovered, mixed with an equal volume of the native loading dye solution (1× TBE, 10%[v/v] glycerol, bromophenol blue, xylene cyanol), and analyzed in 15% native polyacrylamide gel electrophoresis (PAGE) using 0.5× TBE as running buffer (150 V, 40 min). The signals were detected using BAS-MS imaging plate (FUJIFILM) and Typhoon FLA 9000 image analyzer (GE Healthcare).

### Target RNA for RISC-mediated cleavage

A partial GFP fragment amplified by PCR using TI429 and TI431 (Supplemental Table S1) was cloned between SalI and BamHI sites in pSP64-poly(A) vector (Promega). Plasmid linearized by EcoRI was used as template for in vitro transcription with the SP6-scribe standard RNA IVT kit (Cellscript) in the presence of [α-^32^P]-CTP. The transcripts were capped with the ScriptCap m7G Capping System (Cellscript). The products were purified as described in “Preparation of double-stranded RNA.”

### Preparation of double-stranded RNA

Complementary single-stranded RNAs were synthesized by in vitro transcription using the SP6-scribe standard RNA IVT kit (Cellscript) in the presence of [α-^32^P]-CTP. Template DNAs for in vitro transcription were partial *gfp* fragments containing SP6 promoter. The template fragments were amplified by PCR with oligonucleotides listed in Supplemental Table S1 (TI584, TI585, TI586, TI587, TI591, and TI592) and pSP-Flag-GFP as PCR template, and extracted by gel-purification. After in vitro transcription, the reaction mixtures were passed through mini Quick spin RNA columns (Roche), and purified RNA products were extracted by PCI and precipitated by ethanol. An equal amount of complementary RNAs was mixed and annealed as described in the “Small RNAs.”

### dsRNA processing reaction

Unless otherwise indicated, after in vitro translation reaction, the mixtures were incubated with the 15 nM internally ^32^P-labeled 100-bp dsRNA at 25°C for 30 min with the addition of an ATP-regenerating system composed of 0.75 mM ATP, 1 mM MgCl_2_, 20 mg/mL creatine phosphate, and 0.4 mg/mL creatine kinase. The RNA extraction and analysis were performed as described in the “RISC loading reaction.”

### Removal of low-molecular weight molecules from BYL

Ultrafiltration was performed as previously described (Iki et al. 2010) using Vivaspin (molecular weight cutoff 10,000; GE Healthcare). Column purification was performed using PD SpinTrap G-25 (GE Healthcare). In vitro translation reaction mixtures (140 µL) were passed through columns preequilibrated with TR buffer (30 mM HEPES [pH 7.4], 80 mM KOAc, 1.8 mM MgCl_2_, 2 mM DTT, 1 tablet/50 mL complete protease inhibitor [Roche]).

### Nucleotide incorporation by RDR activities

The template ssRNA was synthesized by in vitro transcription using SP6-scribe standard RNA IVT kit (Cellscript), and purified as described in “Preparation of dsRNA.” The ∼100-nt ssRNA fragment is identical to the sense *gfp* fragment used for dsRNA preparation. The ∼1000-nt ssRNA fragment contains sequence corresponding to *A. thaliana* TAS1C. HA-RDR1, HA-RDR2, HA-RDR6, or GFP-HA (control condition) was expressed in BYL by in vitro translation, followed by immunopurification using TR buffer-equilibrated 10 µL anti-HA magnet beads (PIERCE). After the washing step with TR buffer, the magnet beads were incubated at 25°C for 2 h in the 20 µL reaction solution containing 750 nM 100-nt or 300 nM 1000-nt in vitro transcript as template together with 1mM ATP, 1 mM CTP, 1 mM GTP, 0.1 mM UTP, [α-^32^P]-UTP, and 12 mM MgCl_2_. The mixture was used for “dsRNA processing reaction.”

### Immunopurification

To immunopurify TCV P38, the reaction mixtures were incubated with anti-P38 antibody-conjugated protein A magnet beads (Millipore) for 60 min on ice. For antibody conjugation, magnet beads were treated with anti-P38 serum at 1:100 dilution for 3 h on ice, and washed three times with TR buffer. To immunopurify Flag-tagged proteins, the reaction mixtures were incubated with TR buffer-equilibrated anti-Flag M2 magnet beads (Sigma-Aldrich) for 60 min on ice. After incubation, magnet beads were washed three times with TR buffer. Bead-associated proteins were extracted by boiling in WB loading buffer, and analyzed by immunoblotting. Beads-associated RNAs were extracted by adding TE and PCI (1:1 v/v), then analyzed as described above in “RISC loading reaction.”

### Transient expression in *N. benthamiana*

The fragments encoding TCV P38, the mutant derivatives, and CCFV P38 were inserted downstream from Cauliflower mosaic virus 35S promoter on pB7WG2 binary vector. All the pB7WG2-derived constructs were transformed into *Agrobacterium tumefaciens* strain AGLO by electroporation. The leaves of *N. benthamiana* grown at 21°C in the long day condition (16 h of light and 8 h of dark) were infiltrated with *A. tumefaciens*. Before the infiltration, the *A. tumefaciens* cells were incubated for 5 h in the induction buffer (60 mM K_2_HPO_4_, 33 mM KH_2_PO_4_, 8 mM (NH_4_)_2_SO_4_, 2 mM sodium citrate, 0.5 mM MES, 0.4% [v/v] glycerol, and 200 µM acetosyringone), and then resuspended in the infiltration buffer (10 mM MES [pH 5.8], 10 mM MgCl_2_) to a concentration of optical density at 600 nm of 0.4 for *gfp* and 0.1 for *P38* genes. The optimal density at 600 nm was 0.2 for both *gffg* hairpin and *P38* genes. Four days after infiltration, the leaf discs were sampled for pictures, or the leaves were frozen by liquid nitrogen and powdered with mortar. The leaf powder was incubated in the lysis buffer (50 mM Tris–HCl [pH 7.5], 150 mM, NaCl, 2 mM Mg(OAc)_2_, 2 mM DTT, 10% [v/v] glycerol, 0.1% [v/v] NP-40, 1 tablet/50 mL complete protease inhibitor [Roche]), by rotating at 4°C for 30 min. The crude extract was cleared by repeated centrifugation (15,000*g*, 5 min, 4°C), and the supernatant was mixed with an equal volume of 2× WB loading buffer and analyzed by immunoblotting. RNA extraction was performed by mixing the supernatant with TRIzol reagent (Thermo Fisher Scientific). For immunopurification, the supernatant was prepared in TR buffer and incubated with anti-P38 antibody (1:2000) at 4°C for 60 min, followed by the addition of protein A magnet beads (Millipore) and further incubation at 4°C for 60 min. Following procedures are as described in “Immunopurification.”

### Immunoblot analysis

To detect TCV P38 protein, the anti-TCV P38 antibody and the horseradish peroxidase (HRP) conjugated goat anti-rabbit IgG secondary antibody (Invitrogen) was used at 1:10,000 dilution. To detect Flag-tagged P38 and derivative proteins, the monoclonal anti-Flag M2-HRP antibody (Sigma-Aldrich) was used at 1:8000 dilution. The chemiluminescent signals induced by Lumi-Light Western Blotting Substrate (Roche) were detected using X-Ray film (FUJIFILM) and CURIX 60 processor (AGFA).

### RNA gel blot analysis

Total RNA (∼5 µg) was analyzed by 7 M urea-containing 17.5% PAGE using 0.5× TBE as the running buffer (150 V 90 min). RNA was transferred to Hybond-NX nylon membrane (GE), and chemically cross-linked with 1-ethyl-3-(3-dimethylaminopropyl) carbodiimide (Sigma-Aldrich) (Pall and Hamilton 2008). Hybridization was performed with ^32^P-labeled DNA probes in PerfectHyb Plus Hybridization buffer (Sigma). The 5′ end labeling on synthetic DNA oligo probes was performed with [γ-^32^P]-ATP (Hartmann) using T4 Polynucleotide kinase (Thermo Fisher Scientific). The *gffg*- or *GFP*-derived fragments were hybridized with internally ^32^P-labeled DNA probes prepared by the Prime-a-Gene Labeling System (Promega) using [α-^32^P]-dCTP (Hartmann).

## SUPPLEMENTAL MATERIAL

Supplemental material is available for this article.

## Supplementary Material

Supplemental Material
