# Supplementary material for: Biochemical and genetic functional dissection of the P38 viral suppressor of RNA silencing
Source: RNA. 2017 May;23(5):639–54. doi: 10.1261/rna.060434.116 (PMC5393175; doi:10.1261/rna.060434.116)
Supplement: Supplemental Material [file supp_23_5_639__index.html]

Biochemical and genetic functional dissection of the P38 viral suppressor of RNA silencing — Supplemental Material 

# Biochemical and genetic functional dissection of the P38 viral suppressor of RNA silencing

## Supplemental Material

- Supplemental\_Figure\_S1.docx
- Supplemental\_Figure\_S2.docx
- Supplemental\_Figure\_S3.docx
- Supplemental\_Figure\_S4.docx
- Supplemental\_Figure\_S5.docx
- Supplemental\_Figure\_S6.docx
- Supplemental\_Table\_S1.docx
