# Supplementary material for: Biochemical and genetic functional dissection of the P38 viral suppressor of RNA silencing
Source: RNA. 2017 May;23(5):639–54. doi: 10.1261/rna.060434.116 (PMC5393175; doi:10.1261/rna.060434.116)
Supplement: Supplemental Material [file supp_060434.116_Supplemental_Figure_S1.docx]

**
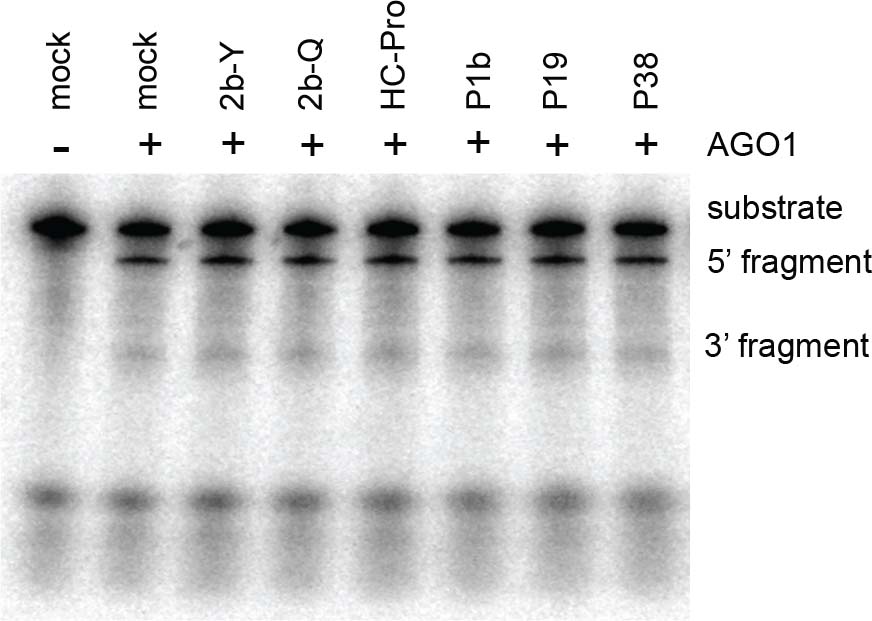
**

**Figure S1. Effect of VSRs on RISC-mediated target RNA cleavage.**

AGO1 was expressed in BYL by *in vitro* translation, and loaded with 5 nM unlabeled 21-nt siRNA duplexes for 60 min. The siRISC-containing solution was mixed (1:1 v/v) with BYL expressing indicated VSR (or mock), and then incubated with 3.5 nM internally ^32^P-labeled target RNA. The 374-nt target RNA is expected to generate 269-nt 5’ fragment and 105-nt 3’ fragments by the endonucleolytic activity of siRISC. The 2b encoded by CMV strain Y was analyzed in the main figure.
