# Supplementary material for: Biochemical and genetic functional dissection of the P38 viral suppressor of RNA silencing
Source: RNA. 2017 May;23(5):639–54. doi: 10.1261/rna.060434.116 (PMC5393175; doi:10.1261/rna.060434.116)
Supplement: Supplemental Material [file supp_060434.116_Supplemental_Figure_S2.docx]

**
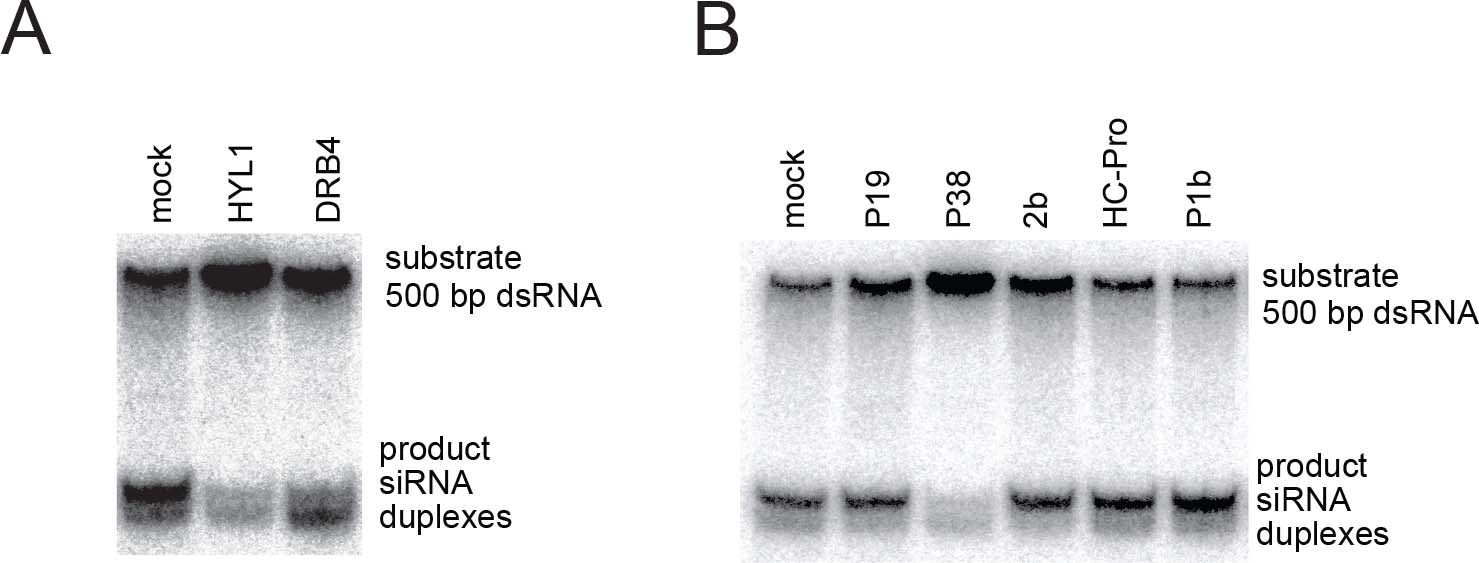
**

**Figure S2. Effect of the expression of DRB proteins or VSRs on siRNA processing.**

**A** Effect of the addition of HYL1 or DRB4 by in vitro translation on the processing activities for 3 nM 500-bp dsRNA.

**B** Effect of the expression of VSRs by in vitro translation on the processing activities for 3 nM 500-bp dsRNA.
