# Supplementary material for: Biochemical and genetic functional dissection of the P38 viral suppressor of RNA silencing
Source: RNA. 2017 May;23(5):639–54. doi: 10.1261/rna.060434.116 (PMC5393175; doi:10.1261/rna.060434.116)
Supplement: Supplemental Material [file supp_060434.116_Supplemental_Figure_S3.docx]

**
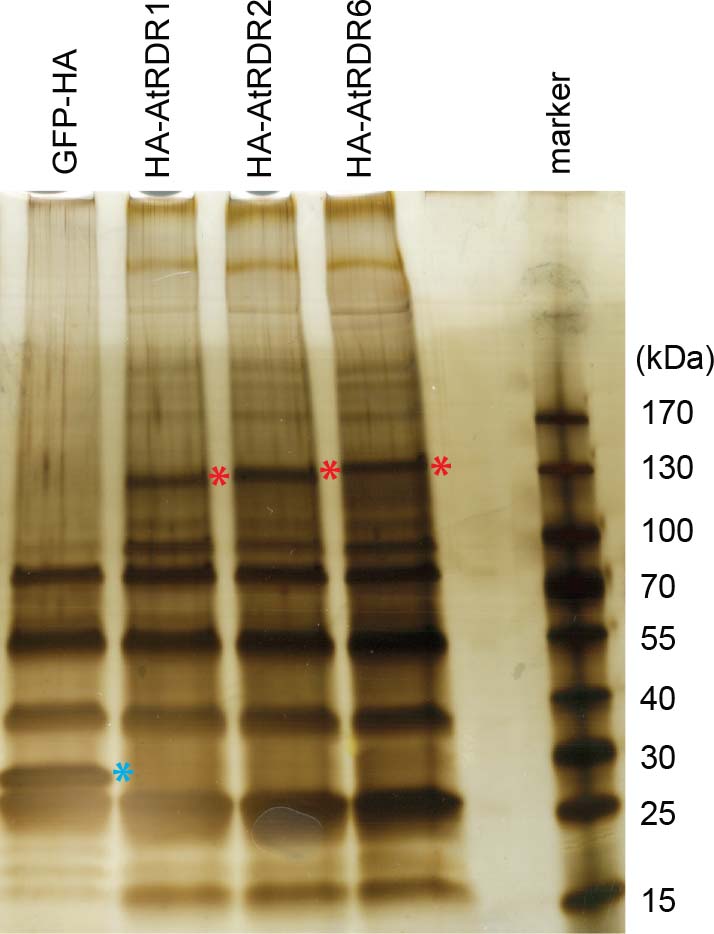
**

**Figure S3. Silver staining image for the fractions immunopurified with anti-HA antibodies**

BYL expressing GFP-HA, HA-RDR6, HA-RDR2, or HA-RDR6 by in vitro translation were incubated with anti-HA antibody-conjugated magnet beads. Immunopurified HA-tagged proteins are indicated by asterisks (red: RDRs, blue: GFP).
