# Supplementary material for: Biochemical and genetic functional dissection of the P38 viral suppressor of RNA silencing
Source: RNA. 2017 May;23(5):639–54. doi: 10.1261/rna.060434.116 (PMC5393175; doi:10.1261/rna.060434.116)
Supplement: Supplemental Material [file supp_060434.116_Supplemental_Figure_S4.docx]

**Figure S4. Phylogenetic analysis of carmovirus P38 proteins**

TCV P38 (ADT78694), CCFV P38 (NP_041887.1), PFBV P38 (ABD93258.1), PLPV P37 (ACJ38486.1), *Calibrachoa mottle virus* P37 (YP_008378655.1), *Angelonia flower break virus* P38 (YP_459964.1), *Hibiscus chlorotic ringspot virus* (ABD48712.1), *Japanese iris necrotic ring virus* P38 (NP_038458.1) are aligned using ClastalW.
