# Supplementary material for: Biochemical and genetic functional dissection of the P38 viral suppressor of RNA silencing
Source: RNA. 2017 May;23(5):639–54. doi: 10.1261/rna.060434.116 (PMC5393175; doi:10.1261/rna.060434.116)
Supplement: Supplemental Material [file supp_060434.116_Supplemental_Figure_S5.docx]

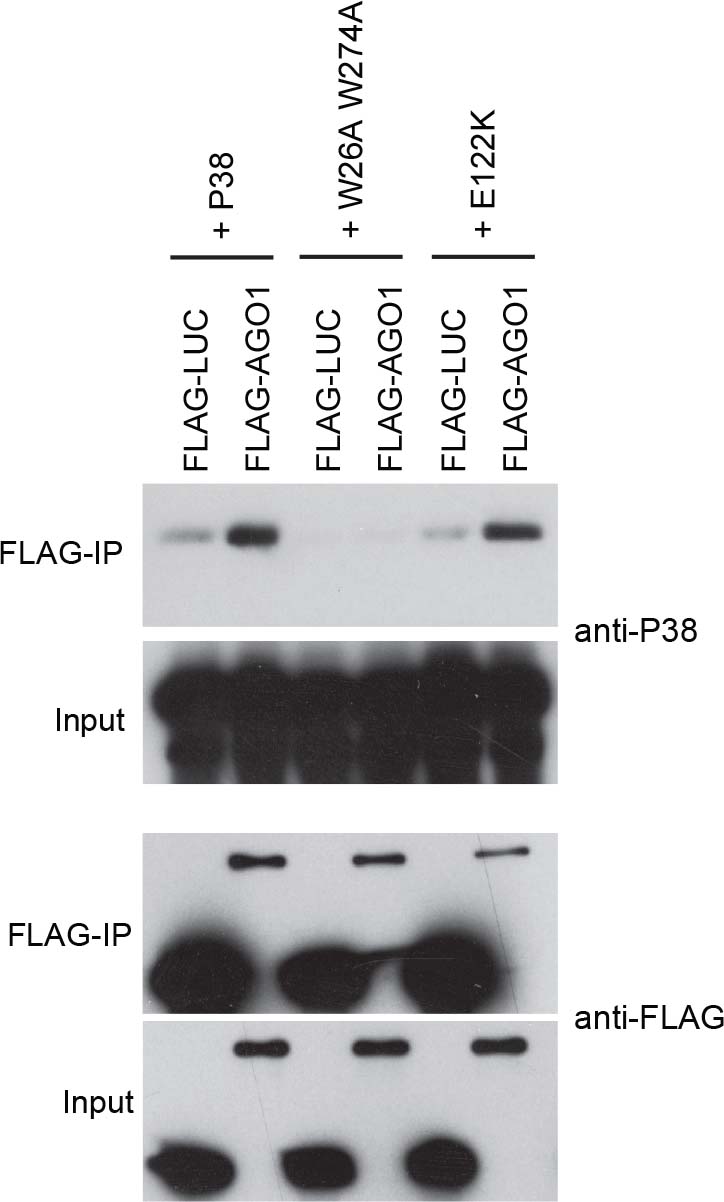


**Figure S5. Physical interaction between AGO1 and TCV P38 or its mutant derivatives in vitro**

Indicated in vitro translation mixtures were mixed (1:1 v/v), and incubated in the addition of ATP-regeneration system as for RISC loading reaction. FLAG-tagged proteins were then immunopurified using EZview Red ANTI-FLAG M2 affinity gel (SIGMA).
