# Supplementary material for: Biochemical and genetic functional dissection of the P38 viral suppressor of RNA silencing
Source: RNA. 2017 May;23(5):639–54. doi: 10.1261/rna.060434.116 (PMC5393175; doi:10.1261/rna.060434.116)
Supplement: Supplemental Material [file supp_060434.116_Supplemental_Figure_S6.docx]

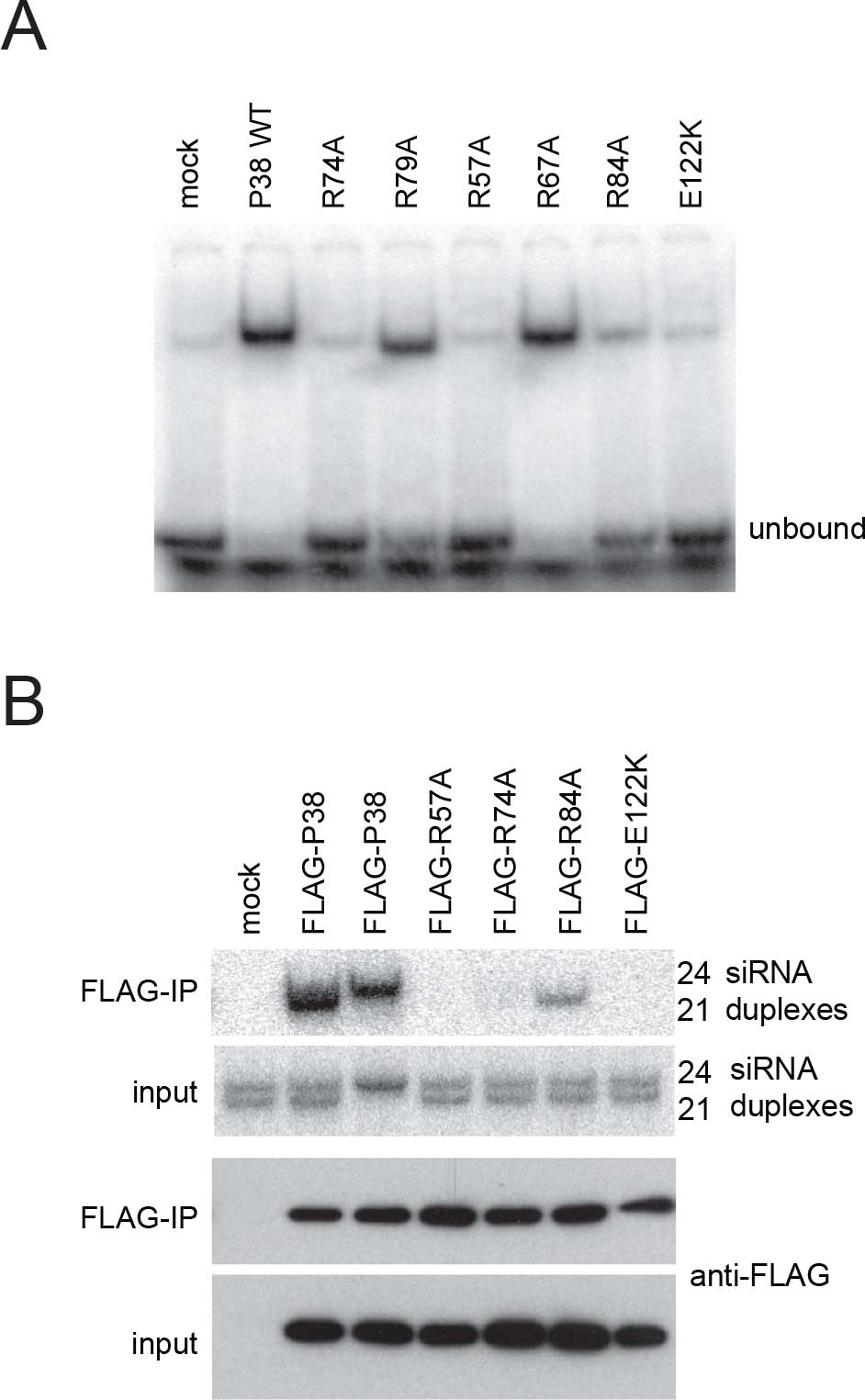


**Figure S6. Interaction between P38 derivatives and siRNA duplexes.**

**A** Gel mobility shift of ^32^P-labeled 21-nt siRNA duplexes induced by TCV P38 and derivatives.

**B** Size-selective purification of siRNA duplexes with FLAG-tagged TCV P38 and the derivatives. In vitro translation mixtures were incubated with ^32^P-labeled 24-nt and 21-nt siRNA duplexes (10 nM each) for 60 min, followed by immunopurification using anti-FLAG antibody.
