# Supplementary material for: Biochemical and genetic functional dissection of the P38 viral suppressor of RNA silencing
Source: RNA. 2017 May;23(5):639–54. doi: 10.1261/rna.060434.116 (PMC5393175; doi:10.1261/rna.060434.116)
Supplement: Supplemental Material [file supp_060434.116_Supplemental_Table_S1.docx]

Table S1. Oligonucleotides used in this study

| Name of oligo | sequence (5'-3') | note |
| --- | --- | --- |
| TI409-P19TBSV-F1 | ACTGACCTGCAGATGGAACGAGCTATACAAGGA | TI409 and TI410 were used to amplify TBSV P19 fragment |
| TI410-P19TBSV-R1 | ACTGACGGATCCCCGGTTTAGCGAGGAACTTCTTTACTCGCTTTCTTTTTCG |  |
|  |  |  |
| TI413-P38TCV-F1 | ACTGACCTGCAGATGGAAAATGATCCTAGAGTC | TI413 and TI414 were used to amplify TCV P38 fragment |
| TI414-P38TCV-R1 | ACTGACGGATCCCCGGTTTAGCGAGGAACTTCTCTAAATTCTGAGTGCTTGCA |  |
|  |  |  |
| TI425-P1bCVYV-F1 | ACTGACGTCGACATGACAATTCATGGATTGCATGC | TI425 and TI426 were used to amplify CVYV P1b fragment |
| TI426-P1bCVYV-R1 | ACTGACGAGCTCCCGGTTTAGCGAGGAACTTCTCTAATAAAAGTCAATTTTATCTTTCTC |  |
|  |  |  |
| TI411-2bCMV-F1 | ACTGACCTGCAGATGGAATTGAACGTAGGTGC | TI412 and TI433 were used to amplify CMV 2b fragment |
| TI433-2bfull-R | ACTGACGGATCCCCGGTTTAGCGAGGAACTTCTTCATCAGAAAGCACCTTCCGCCCATTCGTTACCGGCGAACCAATCTGTATCGTC | |
|  |  |  |
| TI434-HcproPVY-F1 | ACTGACCTGCAGATGGGGGTTATGGATTCAATGG | TI434 and TI436 were used to amplify PVY HC-Pro fragment |
| TI436-HcproPVY-R2 | ACTGACGGATCCCCGGTTTAGCGAGGAACTTCTTTAACCAACTCTATAGTGC |  |
|  |  |  |
| TI556-HC-RLHAAA-F | TACGAAAATGCAGCAGCACCGAATGGGACAAGAAAACT | TI556 and TI557 to were used to introduce AS9mimic mutation on Hc-Pro |
| TI557-HC-RLHAAA-R | CATTCGGTGCTGCTGCATTTTCGTATGCTGAATAGC |  |
|  |  |  |
| TI558-2b-P41A-F | ACAAAAGTGCCAGCGAGAGAGCGCGTTCAAATC | TI558 and TI559 were used to introduce P41A mutation on 2b |
| TI559-2b-P41A-R | TCTCGCTGGCACTTTTGTGACCTCGTTCC |  |
|  |  |  |
| TI560-P1b-RKAA-F | CATGCTTGGCAGCAGCAACTAAGTATGGTGTTGG | TI560 and TI561 were used to introduce R69A K70A mutations on P1b |
| TI561-P1b-RKAA-R | TTAGTTGCTGCTGCCAAGCATGCCAAAGCTGGTTTTG |  |
|  |  |  |
| TI678-P38R74W-F | CCCAGCCTTGGGTCTCTACTGCCAGGGACGGCA | TI678 and TI679 were used to introduce R74W mutation on P38 |
| TI679-P38R74W-R | AGAGACCCAAGGCTGGGTGGTAACCTCGCGGT |  |
|  |  |  |
| TI696-P38R74A-F | CCCAGCCTGCGGTCTCTACTGCCAGGGACGGCA | TI696 and TI697 were used to introduce R74A mutation on P38 |
| TI697-P38R74A-R | AGAGACCGCAGGCTGGGTGGTAACCTCGCGGT |  |
|  |  |  |
| TI680-P38E122K-F | TCATTAAGAAGGCGGCCCAGTATGAAAAATAC | TI680 and TI681 were used to introduce E122K mutation on P38 |
| TI681-P38E122K-R | GCCGCCTTCTTAATGAGCTGGTTGAATGTTC |  |
|  |  |  |
| TI759-R8A-F | CTAGAGTCGCGAAGTTCGCATCTGATGGCGC | TI759 and TI760 were used to introduce R8A mutation on P38 |
| TI760-R8A-R | CGAACTTCGCGACTCTAGGATCATTTTCCA |  |
|  |  |  |
| TI761-R32A-F | TAACCAGCGCACAGAAACAGACCGCCCGCGC | TI761 and TI762 were used to introduce R32A mutation on P38 |
| TI762-R32A-R | GTTTCTGTGCGCTGGTTAGGGTTGACCAGC |  |
|  |  |  |
| TI747-R57A-F | AAGTGACTGCACTGAGTGCTCCGGTGGCCCTTG | TI747 and TI748 were used to introduce R57A mutation on P38 |
| TI748-R57A-R | CACTCAGTGCAGTCACTTTCTGCACAGGTTG |  |
|  |  |  |
| TI749-R67A-F | TTGCCTACGCCGAGGTTACCACCCAGCCTCG | TI749 and TI750 were used to introduce R67A mutation on P38 |
| TI750-R67A-R | TAACCTCGGCGTAGGCAAGGGCCACCGGAGC |  |
|  |  |  |
| TI733-P38-R79A-F | CTACTGCCGCGGACGGCATAACCAGAAGCGG | TI733 and TI734 were used to introduce R79A mutation on P38 |
| TI734-P38-R79A-R | TGCCGTCCGCGGCAGTAGAGACCCGAGGCTG |  |
|  |  |  |
| Ti751-R84A-F | GCATAACCGCAAGCGGTTCTGAACTGATCAC | TI751 and TI752 were used to introduce R84A mutation on P38 |
| TI752-R84A-R | AACCGCTTGCGGTTATGCCGTCCCTGGCAGTAG |  |
|  |  |  |
| TI779-R241A-F | TCAAGAACGCAACTGGCTCAACCAGCGACGC | TI779 and TI780 were used to introduce R241A mutation on P38 |
| TI780-R241A-R | AGCCAGTTGCGTTCTTGAGCTGCACGGTGTAC |  |
|  |  |  |
| TI765-R350A-F | AAGCACTCGCAATTTAGAGAAGTTCCTCGCTA | TI765 and TI766 were used to introduce R350A mutation on P38 |
| TI766-R350A-R | CTCTAAATTGCGAGTGCTTGCCATTTACCCTTTG |  |
|  |  |  |
| TI763-K121A-F | AGCTCATTGCGGAGGCGGCCCAGTATGAAAAAT | TI763 and TI764 were used to introduce K121A mutation on P38 |
| TI764-K121A-R | CCGCCTCCGCAATGAGCTGGTTGAATGTTCC |  |
|  |  |  |
| TI735-P38-E313A-F | CCTGGGAGCAGCCGCAGCAGGTAGTGTCC | TI735 and TI736 were used to introduce E313A mutation on P38 |
| TI736-P38-E313A-R | CTGCGGCTGCTCCCAGGACCGAGAAGTCAG |  |
|  |  |  |
| TI647-FLAG-P38-F | ATGGACTACAAGGATGACGATGACAAGGAAAATGATCCTAGAGTCCG | TI647 and TI648 were used to add FLAG epitope to P38 |
| TI648-FLAG-P38-R | ATCGTCATCCTTGTAGTCCATCTGCAGCCCAAGCTTGTATTC |  |
|  |  |  |
| TI570-DRB1-F1 | ACTGACGTCGACTCTTCGCCGTGCCCTACTAACCT | TI570 and TI571 were used to amplify DNA fragment encoding HYL1(DRB1) by PCR |
| TI571-DRB1-R1 | ACTGACGGATCCTTCTCTTCAATCTGTGAATTACTAC |  |
|  |  |  |
| TI575-DRB4-F1 | ACTGACCTGCAGTTTAGATAGAGATGGATCATGTATACAAAGGTCAA | TI575 and TI576 were used to amplify DNA fragment encodingDRB4 by PCR |
| TI576-DRB4-R1 | ACTGACGGATCCGCATCAAAGATGATGATGTCGTTATGGCTTCACAAGACGATAG |  |
|  |  |  |
| TI599-RDR6-F1 | ACTGACGTCGACGCTTCTATTTCTACCTCGTATCCAG | TI599 and TI600 were used to amplify DNA fragment encoding RDR6 by PCR |
| TI600-RDR6-R1 | ACTGACTCTAGATAACCTTTTAGAGACGCTGAGCAAG |  |
| TI601-RDR6-R0 | CAGAGTGGCCTTACGGCATTAAAC | TI601 was used to prepare RDR6-specific cDNA |
| TI645-HA-RDR6-F | ATGTACCCATACGATGTTCCAGATTACGCTGGGTCAGAGGGAAATATGAAGAAG | TI645 and TI646 were used to add HA epitope to RDR6 |
| TI646-HA-RDR6-R | TGGAACATCGTATGGGTACATTTCTCACAGTGAGATCTTTTTCAATC |  |
|  |  |  |
| TI649-atRDR1-F1 | ACTGACCTGCAGGAGGGAACAGAGATCATTCATCGC | TI649 and TI650 were used to amplify DNA fragment encoding RDR1 by PCR |
| TI650-atRDR1-R1 | ACTGACGGATCCTGTACATCATAAGACATAAGTCAG |  |
| TI651-atRDR1-R0 | ATTTATAGAAATTGCTGATTGCAC | TI651 was used to prepare RDR1-specific cDNA |
| TI659-HAatRDR1-F | ATGTACCCATACGATGTTCCAGATTACGCTGGGAAGACAATTCAAGTGTTTGG | TI659 and TI660 were used to add HA epitope to RDR1 |
| TI660-HAatRDR1-R | TGGAACATCGTATGGGTACATCTTTCTCTATATCAACCGCG |  |
|  |  |  |
| TI653-atRDR2-F1 | ACTGACGGATCCCTCTTCACACTGTCCCCGTCTCTC | TI653 and TI654 were used to amplify DNA fragment encoding RDR2 by PCR |
| TI654-atRDR2-R1 | ACTGACGGATCCGTGTTATATAGGGCAATCAAATGG |  |
| TI655-atRDR2-R0 | GTCCTTTAACTAGCAATTCTACTAC | TI655 was used to prepare RDR2-specific cDNA |
| TI663-HAatRDR2-F | ATGTACCCATACGATGTTCCAGATTACGCTGTGTCAGAGACGACGACGAACCG | TI663 and TI662 were used to add HA epitope to RDR1 |
| TI664-HAatRDR2-R | TGGAACATCGTATGGGTACATGATTAACCCAAGAGAAAGAGAG |  |
|  |  |  |
| TI566-TAS1C-F | ACTGACCTGCAGAAACCTAAACCTAAACGGCT | TI566 and TI567 were used to amplify DNA fragment containing TAS1C sequence by PCR |
| TI567-TAS1C-R | ACTGACGGATCCATTTCACTTTACGATGTGGT |  |
|  |  |  |
| TI584-ds98/100-98F-SP6 | GTATCATACACATACGATTTAGGTGACACTATAGAAAACTACCTGTTCCATGGCCAAC | TI584 and TI585 to amplify ~100-bp gfp connected to SP6 promoter sequence to synthesize sense strand partial gfp ssRNA by in vitro transcription |
| TI585-ds98/100-98R | GAAGTCGTGCCGCTTCATATG |  |
|  |  |  |
| TI586-ds98/100-100R-SP6 | GTATCATACACATACGATTTAGGTGACACTATAGAAGTCGTGCCGCTTCATATGATC | TI586 and TI587 to amplify ~100-bp gfp connected to SP6 promoter sequence to synthesize antisense strand partial gfp ssRNA by in vitro transcription |
| TI587-ds98/100-100F | TGGAAAACTACCTGTTCCATG |  |
|  |  |  |
| TI591-ds-s510R | GAAGGACCATGTGGTCTCTCTTTTCG | TI584 and TI591 to amplify ~500-bp gfp connected to SP6 promoter sequence to synthesize sense strand partial gfp ssRNA by in vitro transcription |
|  |  |  |
| TI592-ds-as512R-SP6 | GTATCATACACATACGATTTAGGTGACACTATAGAAGGACCATGTGGTCTCTCTTTTCG | TI587 and TI592 to amplify ~500-bp gfp connected to SP6 promoter sequence to synthesize antisense strand partial gfp ssRNA by in vitro transcription |
|  |  |  |
| NP19-P38 (attB1) | GGGGACAAGTTTGTACAAAAAAGCAGGCTATGGAAAATGATCCTAGAGTCCG | NP19 and TI677 to amplify fragments encoding TCV P38 or the derivatives, franked by attB1 and attB2 sequences required for the insertion in pDONR221 entry vector |
| TI677-P38(attB2) | GGGGACCACTTTGTACAAGAAAGCTGGGTACTAAATTCTGAGTGCTTGCCATT |  |
| TI725-P38CCFV-attB1-F | GGGGACAAGTTTGTACAAAAAAGCAGGCTACCATGGCAATTAAGGAGGACCCA | TI725 and TI726 to amplify fragment encoding CCFV P38, franked by attB1 and attB2 sequences required for the insertion in pDONR221 entry vector |
| TI726-P38CCFV-attB2-R | GGGGACCACTTTGTACAAGAAAGCTGGGTATTACAGGCGAGACACAGACCA |  |
